# Papillomavirus Genomes Associate with BRD4 to Replicate at Fragile Sites in the Host Genome

**DOI:** 10.1371/journal.ppat.1004117

**Published:** 2014-05-15

**Authors:** Moon Kyoo Jang, Kui Shen, Alison A. McBride

**Affiliations:** 1 Laboratory of Viral Diseases, National Institute of Allergy and Infectious Diseases, National Institutes of Health, Bethesda, Maryland, United States of America; 2 Bioinformatics and Computational Biosciences Branch, National Institute of Allergy and Infectious Diseases, National Institutes of Health, Bethesda, Maryland, United States of America; University of Wisconsin-Madison, United States of America

## Abstract

It has long been recognized that oncogenic viruses often integrate close to common fragile sites. The papillomavirus E2 protein, in complex with BRD4, tethers the viral genome to host chromatin to ensure persistent replication. Here, we map these targets to a number of large regions of the human genome and name them Persistent E2 and BRD4-Broad Localized Enrichments of Chromatin or PEB-BLOCs. PEB-BLOCs frequently contain deletions, have increased rates of asynchronous DNA replication, and are associated with many known common fragile sites. Cell specific fragile sites were mapped in human C-33 cervical cells by FANCD2 ChIP-chip, confirming the association with PEB-BLOCs. HPV-infected cells amplify viral DNA in nuclear replication foci and we show that these form adjacent to PEB-BLOCs. We propose that HPV replication, which hijacks host DNA damage responses, occurs adjacent to highly susceptible fragile sites, greatly increasing the chances of integration here, as is found in HPV-associated cancers.

## Author Summary

Papillomavirus cause persistent, but mostly self-limiting, infections of the host epithelium. However, a subset of oncogenic papillomaviruses is the causative agent of certain human cancers. In persistent infection the viral genomes are tethered to host chromosomes to maintain and partition the extrachromosomal viral genomes to daughter cells. However, in cancers viral DNA is often found integrated close to common fragile sites, regions prone to breakage, amplification and deletion. We show that the viral E2 and cellular BRD4 proteins are associated with fragile regions of the human genome and nucleate viral replication foci at these sites. This is a resourceful strategy for a virus that uses the host DNA damage response to amplify viral DNA. However, the outcome may be increased accidental integration of viral DNA, which in the case of the oncogenic viruses can promote carcinogenesis.

## Introduction

Papillomaviruses are an ancient group of viruses that establish a persistent infection in the host epithelium. To maintain such a long-term infection, the E2 protein from a subset of papillomaviruses binds to the viral genome and tethers it to the host chromosomes [1]–[3]. The bromodomain protein, BRD4, binds to mitotic chromosomes with E2 [4], [5], is essential for regulation of viral transcription [6]–[9] and is recruited to early viral replication foci [10], [11]. BRD4 is a mitotic chromosome-associated protein [12] that interacts with acetylated histone tails [13] and is a key regulator of the pTEF-b elongation factor [14]. There has been a recent explosion of data as BRD4 has been implicated in regulation of cell cycle, mitotic memory, transcription of MYC and regulation of viral gene expression [15]–[19]. BRD4 is highly enriched at super-enhancers that maintain expression of oncogenes in tumors [20] and is a promising therapeutic target for a number of cancers [21].

Most HPV infections result in benign lesions, but several are oncogenic and the causative agents of human cancer [22]. Almost all cervical cancer is associated with HPV infection, and oncogenic HPVs are responsible for many anal, penile, vaginal and oropharyngeal cancers [23]. The HPV genome is found integrated into the host genome in over 80% cancers and this promotes malignant progression. The integration event is accidental, but the resulting deregulation of expression of the E6 and E7 oncogenes gives cells a selective growth advantage [24]. There is a predilection for integration within the vicinity of fragile sites [25], [26].

Papillomaviruses are adept at hijacking host functions and induce a host DNA damage response (DDR) in nuclear foci, resulting in an influx of repair factors that the virus exploits to amplify its own DNA [11], [27]–[31]. We show that the HPV E2 protein binds with BRD4 to regions that are highly susceptible to replication stress and overlap many common fragile sites. Common fragile sites are hypersensitive to DNA damage and their replication is often incomplete in the G2 phase of the cell cycle [32]. Thus, they represent a vulnerable and very clever target for papillomavirus replication. Furthermore, replication adjacent to fragile sites may explain the high incidence of integration of oncogenic HPV genomes at these loci.

## Results

### HPV1 E2 binds to broad regions of human mitotic chromatin

**Figure 1:**
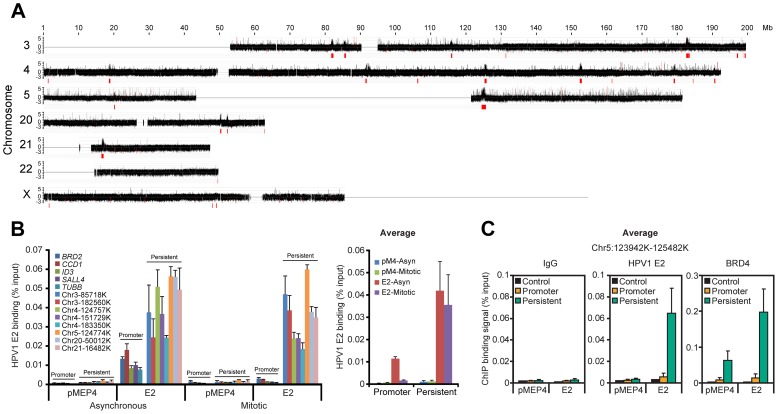
HPV1 E2 binds to broad regions of mitotic chromatin. **A.** ChIP-chip binding profile of E2 on a subset of human chromosomes. E2-bound mitotic chromatin was hybridized to microarray chips containing the chromosomes shown. The Y-axis is a scaled log2-ratio of bound to input signal. Large chromatin regions enriched for E2 binding were identified as described in Methods. They are indicated in red underneath the signal map and in Table S2. Chromosomes 18 and 19 were also analyzed but showed no binding peaks and are not shown. **B.** E2 binding to the large peaks persists throughout the cell cycle. Chromatin was isolated from asynchronous and mitotic fractions from C-33 cells containing empty vector (pMEP4 or pM4) or C-33-1E2 cells (E2) and analyzed by ChIP and Q-PCR using primers (Table S9) for broad E2 binding regions (persistent: throughout the cell cycle) or promoter regions previously shown to bind E2/BRD4 (only in interphase) [34]. Average E2 binding levels were calculated from two independent experiments for five promoter regions and eight broad E2 binding regions. **C.** Average BRD4 and E2 binding levels on mitotic chromatin to five sites within a broad E2 binding region, chr5:123,942,100–125,482,100 in the absence (pMEP4 empty vector) or presence (E2) of E2 expression. E2 and BRD4 binding was analyzed by ChIP using Q-PCR with the primers described in Table S9 and Figure S1C. Average E2 and BRD4 binding levels and STDEV are presented as calculated from four promoter regions (BRD2, CCD1, SALL4, TUBB) and five binding sites within a broad E2 binding peak in Chr5 (See Figure S1 for complete data).

Many papillomavirus E2 proteins bind readily to host mitotic chromosomes with the BRD4 protein [9]. To identify the targets of these E2 proteins we analyzed chromatin binding sites of HPV1 E2, a protein that binds BRD4 and host chromosomes with high affinity. In a natural infection E2 levels range from almost undetectable in basal cells to fairly high levels in differentiated cells [33]; thus we were careful to titrate E2 to low, but detectable, levels for the experiments presented (Figure S1A and S1B). Chromatin was prepared from mitotic C-33 cells expressing HPV1 E2 (C-33-1E2), and analyzed by ChIP-chip analysis for binding to a portion of the human genome (chromosomes 3, 4, 5, 18, 19, 20, 21, 22 and X). We have previously shown by ChIP-chip analysis of 5 kb promoter regions that E2 and BRD4 bind to active promoters in interphase C-33 cells [34]. In the present study we used whole genome tiling arrays to study E2 and BRD4 binding. As shown in Figure 1A, in mitosis E2 was observed to bind to a few extremely broad peaks on several chromosomes. These peaks ranged in size from several hundred Kb to >1 Mb and, for the most part, overlapped coding regions. Two detailed examples of the genomic regions covered by the peaks are shown in Figure S1C.

The mitotic E2 binding peaks were further validated by conventional ChIP assays (Figure 1B) with primers selected from eight of the peaks indicated in Figure 1A. E2 binding to these regions was strong in both asynchronous and mitotic cells, showing that it persisted throughout the cell cycle, consistent with the concept that E2 partitions the viral genome by linking it to mitotic chromosomes [1]. The levels of E2 bound to the broad mitotic regions were several-fold higher than those bound to active promoter regions. Furthermore, the levels of E2 bound to promoters dropped to almost background levels in mitotic cells (Figure 1B), consistent with cessation of transcription and displacement of most transcription factors from promoters in mitosis [35].

Since E2 binds to mitotic chromosomes in complex with BRD4 [4], [5], [7] we carried out ChIP assays to determine whether BRD4 bound the same regions of mitotic chromatin. As shown in Figure S1D, S1E, and S1F (and summarized in Figure 1C) BRD4 bound to five sites selected from an E2 positive region from chromosome 5, even in the absence of E2. However, expression of E2 increased BRD4 binding at least two fold, consistent with the stabilization of BRD4 binding by E2 [7]. In contrast HPV31 E2, which does not stabilize binding of BRD4 to chromatin [9], had little effect on the binding of BRD4 to mitotic chromatin (data not shown). Figure S1G shows a comparison of the size of these broad regions compared to promoter binding of BRD4 and E2 that we had detected previously using promoter microarrays. As we show in more detail below, E2 and BRD4 bind together to these exceptionally large regions of mitotic chromatin that likely correspond to the mitotic chromatin tethering target used by papillomaviruses for genome partitioning. Thus, we have named these regions Persistent E2 and BRD4-Broad Local Enrichments of Chromatin, or PEB-BLOCs.

### The cellular protein, BRD4 binds to PEB-BLOCs with HPV1 E2

**Figure 2:**
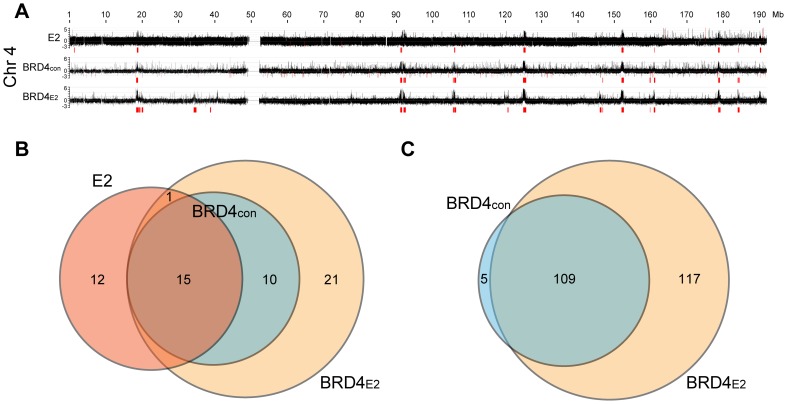
Profile of BRD4 binding to human chromosomes in C-33-1E2 cells. E2 expression was induced in C-33-1E2 cells (and E2 negative control cells) and chromatin was isolated with an anti-BRD4 antibody (C-term) and hybridized to 2.1M human whole genome arrays (NimbleGen). BRD4_CON_ represents negative control C-33 cells and BRD4_E2_ represents C-33 cells expressing HPV1 E2. **A.** The BRD4 binding profiles are shown for chromosome 4 (the complete data set is in Figure S2). The data is aligned with the E2 binding profile for chromosome 4 from Figure 1A. Broad regions of enriched binding were defined computationally and are shown in red (the coordinates are listed in Table S2). **B.** Regions enriched for E2 binding (E2), and BRD4 binding in absence (BRD4_CON_) and presence of E2 (BRD4_E2_), were analyzed and computationally identified in a subset of human chromosomes (chromosome 3, 4, 5, 21, 22, and X) as described in Methods. The overlap between the enriched regions was further calculated as described in Methods and is represented in the Venn diagram. **C.** Broad enriched regions of BRD4 binding in the absence (BRD4_CON_) and presence of E2 (BRD4_E2_) were identified computationally for the entire human genome (Figure S2). The overlap between the enriched regions was calculated as described in Methods and is represented in the Venn diagram.

In C-33-1E2 cells, E2 colocalizes with BRD4 in approximately 50 punctate speckles on mitotic chromosomes (data not shown) and so we extended the pilot experiment described above to analyze BRD4 binding in the entire human genome. BRD4 binding was analyzed by ChIP-chip using whole genome arrays and the BRD4 binding profile is shown in Figure 2A (for chromosome 4) and S2 (for the entire genome). Almost all chromosomes showed large peaks similar in size to, and overlapping with, the E2 peaks identified in the subset of chromosomes shown in Figure 1A. A visual inspection showed that approximately 50 broad BRD4 binding regions were detectable on C-33 mitotic chromatin in the entire genome and about 100 regions were detected in the presence of E2 (Figure S2). Therefore, BRD4 binds to some PEB-BLOCs in the absence of E2, but E2 enhances the BRD4 binding signal. In contrast, BRD4 is only detected on mitotic chromosomes by immunofluorescence in the presence of E2 [7]. This likely reflects differences in sensitivity between the techniques. We have shown previously that the dimerization property of E2 increases the ability of E2-BRD4 complexes to bind mitotic chromosomes, most likely by promoting the formation of higher order complexes [36]. The genomic localization and characteristics of 53 of the strongest PEB-BLOCs identified by visual inspection are listed in Table S1.

We computationally defined and identified the enriched binding regions for E2 and BRD4 (shown in red in Figure 2A and S2). The best algorithm was able to identify all of the visually identified binding peaks, with the exception of one on chromosome 20 (Chr20-P3 in Table S1). Using this algorithm, the overlap between E2 and BRD4 binding regions was calculated, as defined in Methods. Figure 2B shows the overlap among the three binding profiles for chromosomes 3, 4, 5, 20, 21, 22 and X (only a subset of chromosomes was analyzed for binding in these experiments). There was a complete overlap between the BRD4 binding regions in control and E2 expressing cells and >50% overlap with the E2 binding enriched regions and BRD4 binding regions. The overlap with the E2 binding enriched regions is underestimated because of the different resolution of the microarray chips used for the E2 and BRD4 binding studies. However, as can be seen visually in Figure 2A, there is a substantial overlap in the major binding peaks. Figure 2C shows the overlap of computationally defined BRD4 enriched regions, in the presence and absence of E2 expression, for all human chromosomes. Therefore, many PEB-BLOCs exist even without E2 expression and E2 stabilizes and increases Brd4 binding to a subset of PEB-BLOCs. Presumably, different stages of the viral life cycle the levels of E2 would determine which PEB-BLOCs were highly occupied by E2 and BRD4.

### BRD4, and its ability to bind acetylated chromatin, is essential for persistent HPV1 E2 binding

**Figure 3:**
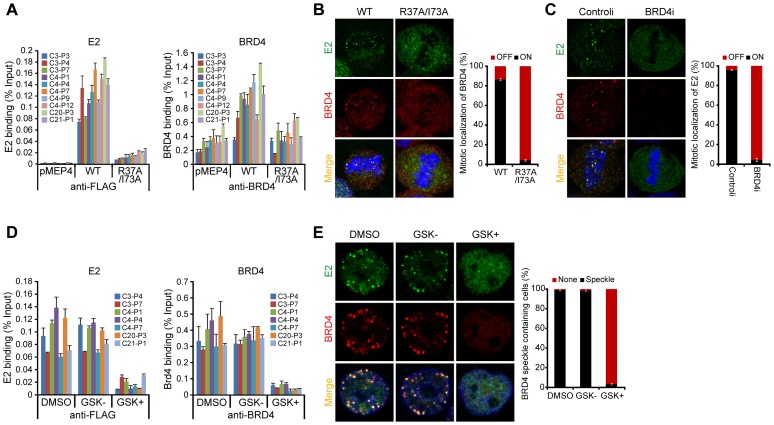
BRD4 is essential for persistent HPV1 E2 binding to host mitotic chromatin. **A.** E2 expression was induced in asynchronous C-33 cells expressing either wild-type or R37A/I73A E2 proteins. Chromatin was isolated with FLAG antibodies (against E2) or with BRD4 antiserum. ChIP DNA was quantitated by Q-PCR using primers specific for the PEB-BLOCs listed. Average values and STDEV were calculated from two independent experiments. **B.** The location of E2 wild-type or E2 R37A/I73A (green) and BRD4 (red) as detected by immunofluorescence. Cellular DNA is counterstained with DAPI in blue. Approximately 50 mitotic cells were analyzed for E2 and BRD4 chromosomal speckles. Average values and STDEV were calculated for three independent experiments. **C.** C-33-1E2 cells were treated with BRD4 siRNA for 3 days and stained for E2 (green), BRD4 (red) and cellular DNA (blue). Approximately 50 mitotic cells were analyzed for E2 and BRD4 chromosomal speckles. Average values and STDEV were calculated for three independent experiments. **D.** C-33 cells expressing HPV1 E2 were treated with DMSO, GSK525762^+^ (GSK+), or GSK525762^−^ (GSK−), for 24 h and E2 expression was induced for 4 h before fixation. Chromatin was isolated with FLAG M2 or with BRD4 immune serum. ChIP DNA was quantitated by Q-PCR using primers specific for the PEB-BLOCs shown. Average values and STDEV were calculated from two independent experiments. **E.** C-33-1E2 cells were treated with DMSO, GSK525762^+^ (GSK+), or GSK525762^−^ (GSK−), for 24 h and E2 expression was induced before fixation. Cells were stained for E2 (green), BRD4 (red) and cellular DNA (blue). Greater than 50 interphase cells were analyzed for E2 and BRD4 colocalization. Average values and STDEV were calculated for three independent experiments.

Two residues in the transactivation domain of E2 (R37 and I73) are essential for interaction with BRD4 [5], [37]. Therefore, we analyzed binding of an E2 R37A/I73A mutated protein to PEB-BLOCs by ChIP. Wild-type E2 and R37A/I73A E2 were expressed at equivalent levels and had no effect on the levels of BRD4 (data not shown). Both wild-type E2 and BRD4 bound strongly to PEB-BLOC regions in asynchronous cells (Figure 3A) and while BRD4 bound to most PEB-BLOCs in the absence of E2, binding was about two fold higher in the presence of E2. However, E2 R37A/I73A was minimally recruited onto and did not augment BRD4 binding to PEB-BLOCs. While BRD4 and E2 colocalize as distinct speckles on mitotic chromosomes, in cells expressing the R37A/I73A protein, neither E2 nor BRD4 was detected on chromosomes (Figure 3B). Therefore the interaction with BRD4 is essential for E2 binding to PEB-BLOCs, but in turn E2 stabilizes the binding of BRD4 to these regions.

To confirm the requirement for BRD4 in E2 binding to mitotic PEB-BLOCs, BRD4 gene expression was downregulated with siRNA. In the absence of BRD4, E2 no longer bound to mitotic chromosomes (Figure 3C) or colocalized in speckles with BRD4 in the nucleus of interphase cells (data not shown). Small molecule inhibitors such as GSK525762A+ interfere with binding of the specific bromodomains of the family of BET proteins (bromodomain plus extraterminal domain) to their acetylated target [38]. In cells treated with GSK525762A^+^, neither E2, nor BRD4, could be detected bound to PEB-BLOC regions by ChIP (Figure 3D). Likewise, E2 and BRD4 speckles were no longer observed in the nuclei of interphase cells (Figure 3E) or on mitotic chromosomes (data not shown) after GSK525762A^+^ treatment. Therefore, E2 binding to PEB-BLOC regions is dependent on BRD4 and its interaction with acetylated histones.

### PEB-BLOCs have a distinctive pattern of histone modification

**Figure 4:**
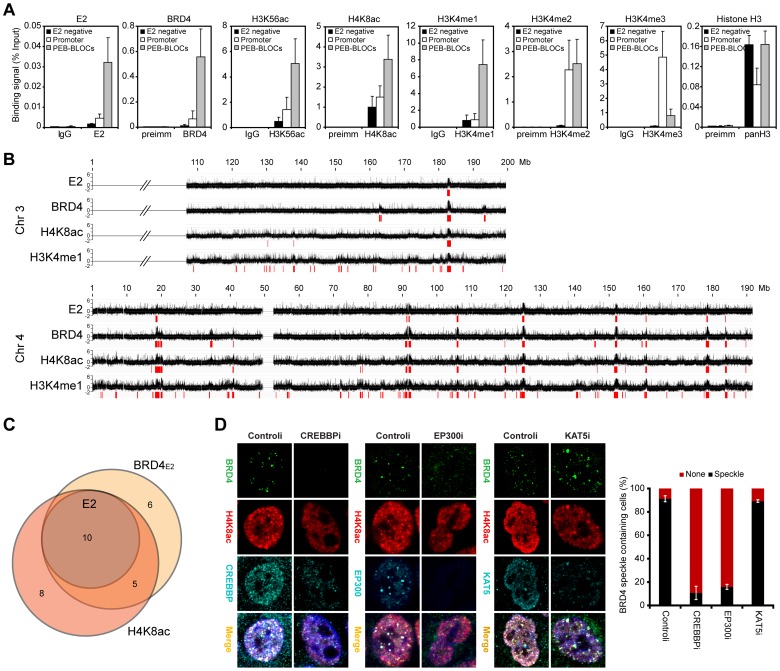
Persistent E2 binding correlates with histone acetylation through CREBBP/EP300 HAT activity. **A.** Mitotic chromatin was isolated from C-33-1E2 cells and immunoprecipitated with control serum or specific antibodies to E2, BRD4, H3K56ac, H4K8ac, H3K4me1, H3K4me2, H3K4me3, and histone H3. ChIP DNA was analyzed by Q-PCR for specific PEB-BLOC regions (listed in Table S9). Average values and STDEV are shown for three independent experiments on four non-E2 binding regions, four active promoters, and six PEB-BLOCs. **B.** ChIP-chip analysis of E2, BRD4, H4K8ac, and H3K4me1 binding in C-33-1E2 cells. Chromatin was prepared from asynchronous cells and isolated using antibodies against E2, BRD4, acH4K8, and H3Kme14. ChIP DNA was hybridized to one HD microarray chip (Nimblegen). The binding profile for E2, BRD4, and histones on chromosome 3 and 4 is shown. Broad regions of enriched binding were defined computationally and are shown in red and are listed in Table S4. **C.** Venn diagram showing the overlap among the enriched binding regions defined in B. **D.** C-33-1E2 cells were treated with CREBBP, EP300 or KAT5 siRNA for 3 days. Cells were stained by immunofluorescence for anti-BRD4 (green), anti-H4K8ac (red), cellular DNA (blue), and anti-CREBBP, -EP300, or -KAT5 antibodies (cyan). The bar chart to the right shows quantification of BRD4 speckle formation in these Interphase cells were analyzed for. Average values and STDEV were calculated for three independent experiments (75–150 cells counted per experiment).

To further investigate the nature of PEB-BLOCs, histone modifications were analyzed by ChIP (Figures 4A and S3) using the primers listed in Table S9. PEB-BLOCs were highly acetylated at positions K9, K14, K18, K23, K27, K56, K9/14, and K9/18 in histone H3 and K5, K8, K12, and K5/8/12/16 in histone H4. E2-BRD4 bound promoter regions showed higher acetylation levels than E2-negative regions, but the acetylation status of PEB-BLOCs was consistently several-fold higher than in active promoter regions, which are already acetylation-rich. Therefore, PEB-BLOCs are highly acetylated at many positions, consistent with the ability of BET inhibitors to abolish E2 and BRD4 binding. Histone methylation, especially of H3K4, is also associated with active chromatin [39]. PEB-BLOCs have consistently high H3K4me1 and H3K4me2, but low H3K4me3. Conversely, promoter regions had high H3K4me2 and H3K4me3, but low H3K4me1 (Figure 4A).

To validate these findings, we performed ChIP-chip analysis for binding of E2, BRD4, H4K8ac, and H3K4me1 on a subset of the genome (chromosome 4 and part of chromosome 3). Each PEB-BLOC overlapped with prominent peaks of H4K8ac and H3K4me1 modification (Figure 4B and 4C). Therefore, PEB-BLOCs contain highly acetylated histones and high levels of H3K4me1, a pattern similar to that described for enhancers [39]. Notably, as shown in Figure 4C, between 65% and 71% of H4K8ac and BRD4 broad enriched regions overlapped and the E2 bound regions were contained completely within this overlap. All E2 bound peaks also completely overlapped with enriched regions of H3K4me1.

To confirm these findings, mitotic and interphase C-33-1E2 cells were analyzed for global histone modification patterns by immunofluorescence (Figure S4 and data not shown). E2-BRD4 speckles colocalized with acH4K8 and acH3K56 on mitotic chromosomes, and were also highly enriched in H3K4me1 and H3K4me2, but not H3K4me3. The E2-BRD4 speckles observed in interphase nuclei also showed an enrichment of acH4K8, acH3K56, H3K4me1 and H3K4me2.

### CREBBP/EP300 HAT activity is responsible for histone acetylation of PEB-BLOCs

To ascertain the histone acetyl transferase (HAT) responsible for acetylation of PEB-BLOCs, we downregulated expression of EP300, CREBBP and KAT5 by siRNA treatment. In control cells, BRD4 speckles colocalized with H4K8ac, CREBBP and EP300. However, siRNA downregulation of CREBBP or EP300 resulted in a great reduction in the appearance of BRD4 speckles as well as the focal regions of histone acetylation in the nucleus (Figure 4D). In contrast, KAT5 only partially colocalized with BRD4 speckles and downregulation of KAT5 had no effect on the acetylation or localization of BRD4 to PEB-BLOCs. Therefore, CREBBP and EP300 are both recruited to PEB-BLOCs where they acetylate histones, thus providing binding sites for E2 and BRD4. Notably, E2 proteins interact with CREBBP/EP300 [40] and this could enhance the formation and development of PEB-BLOCs in a natural infection. However, in C-33 cells these regions are already genetically unstable and highly acetylated, and acetylation is not obviously increased by E2. Regions of chromatin that are methylated on H3K4 show highly dynamic acetylation mediated by CREBBP/EP300, while H3K4 methylation remains more stable [41]. This is consistent with the histone modifications of PEB-BLOCs and the requirement for CREBBP/EP300.

### Individual alleles of many PEB-BLOCs show differential BRD4 binding and asynchronous DNA replication

**Figure 5:**
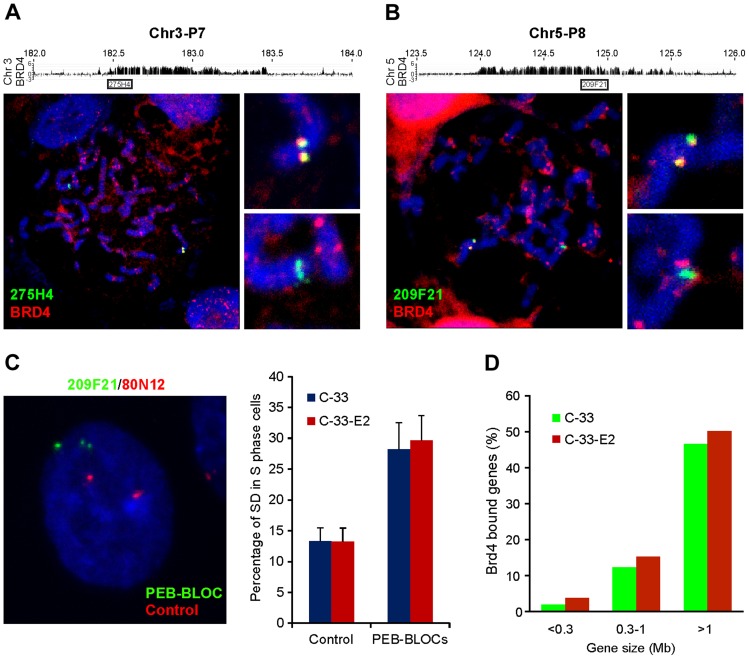
PEB-BLOCs share properties with fragile sites and are targets for chromosomal deletion. **A, B.** Chromosomes were spread from mitotic C-33-1E2 cells induced for E2 expression. BRD4 speckles were identified by immunofluorescence, fixed, and hybridized with PEB-BLOC specific FISH probes (107E21, 209F21, 274H4, or 365P10). A representative example is shown for probes 275H4 (A, Chr3-P7) and 209F21 (B, Chr5-P8). The BRD4 signal is red and the FISH signal is green. **C.** C-33-1E2 cells, induced for E2 expression were analyzed with specific FISH probes. PEB-BLOCs (209F21, 90I21, 274H4, 365P10, 1062A20; green) and control regions (80N12 and 379K17; red). Approximately 50 S-phase cells were analyzed for the SD pattern (one single, one double dot) FISH signals for each PEB-BLOCs or control region. This SD pattern represents asynchronous replication at the allele being examined. Average values and STDEV were calculated for two independent experiments on two control regions (80N12 and 379K17), and 4 PEB-BLOCs (Chr3-P7, Chr5-P8, Chr10-P4, Chr12-P3). **D.** Correlation between gene size and overlapping PEB-BLOC. The overlap was calculated for a region encompassing the gene body plus 5 kb upstream.

To verify that BRD4 nuclear speckles correspond to the regions identified by ChIP-chip, we performed combined IF-FISH with a BRD4 antibody and FISH probes for PEB-BLOCs. In many cases, the BRD4 speckles colocalized with only one of the two PEB-BLOC FISH signals (Figure S5). BRD4 speckles were often observed as doublets on one chromosome, which in mitotic cells colocalized with a similar doublet of FISH signal (Figure 5A and 5B). In contrast, the second PEB-BLOC allele was detected as a condensed FISH signal that didn't colocalize with BRD4, indicating that BRD4 binds to PEB-BLOCs on one allele on mitotic chromosome.

Analysis of the PEB-BLOC FISH signals in interphase cells revealed that the two alleles often replicated at different times. When this occurred, the early replicating allele was observed as a doublet FISH signal, while the late replicating allele was a single FISH signal. When these exist in the same nuclei due to asynchronous replication they are termed SD (singlet-doublet) FISH signals (Figure 5C). We calculated the rate of asynchronous DNA replication for loci corresponding to PEB-BLOCs and non-PEB-BLOCs by counting the number of SD FISH signals in individual nuclei (Figure 5C). Non-PEB-BLOC regions, displayed an SD pattern in ∼12% S-phase cells, as previously reported [42]. In contrast, the SD pattern was present in ∼30% PEB-BLOCS. There was no difference in the percentage of SD signals in control C-33 or C-33-1E2 cells, showing that this is an inherent property of the cells and not due to E2 expression. Notably, asynchronous replication is also a property of common fragile sites [43].

### Most PEB-BLOCs are actively transcribed and often contain very large genes

PEB-BLOCs span large chromosomal regions, which mostly contain annotated genes (Table S1). To determine whether these genes were transcriptionally active, RNA was prepared from control C-33 and C-33-1E2 cells and analyzed by microarray gene expression analysis (data not shown). This showed that most genes located in the PEB-BLOCs were transcribed at low to moderate levels. To determine whether there was additional transcription (perhaps non-mRNA) from apparently non-coding regions, we conducted RNA seq analysis (available at GEO: GSE52367). This confirmed that most PEB-BLOC genes were transcriptionally active, and also identified ten long (>0.2 Mb), previously un-annotated, genes in these regions. These novel genes are shown, along with RNA seq signals for 53 of the strongest PEB-BLOCs in Table S1.

About 35 cellular genes were differentially regulated by E2 expression in both microarray and RNA seq analysis. However, these genes were not associated with PEB-BLOCs and have no obvious connection to E2 function.

There has been reported to be a strong correlation between transcription of very long genes and the expression of fragile sites resulting from a conflict in transcriptional and replication machineries [32]. To date, there are 56 annotated human genes that are >1 Mb and another 219 that are between 0.5–1 Mb long. In the 53 strong PEB-BLOC loci listed in Table S1, there are ten known genes >1 Mb and 19 genes >500 kb. Therefore, there is a vast enrichment of long genes in the PEB-BLOC regions. This calculation does not include the transcriptionally active long segments in PEB-BLOCs that contain unknown genes. Figure 5D shows the size range of genes that overlap PEB-BLOCs.

### PEB-BLOCs are associated with common fragile sites

Many of the properties described above for PEB-BLOCs are also attributes of common fragile sites. These sites are genetically unstable (reviewed in [44]) and are common sites of viral genome integration [25]. Like PEB-BLOCs, common fragile sites often replicate asynchronously, have monoallelic expression and contain large genes [32]. Fragility can arise because of a conflict between transcription and replication of very long genes as a paucity of replication initiation sites can result in failure to complete replication before mitosis [45]. We compared the location of PEB-BLOCs with mapped common fragile sites in the human genome (retrieved from HUGO, www.genenames.org) and found that a subset of visually identified strong PEB-BLOCs (22 out of 53) contain 25 known fragile sites in the same chromosomal band (Table S1).

**Figure 6:**
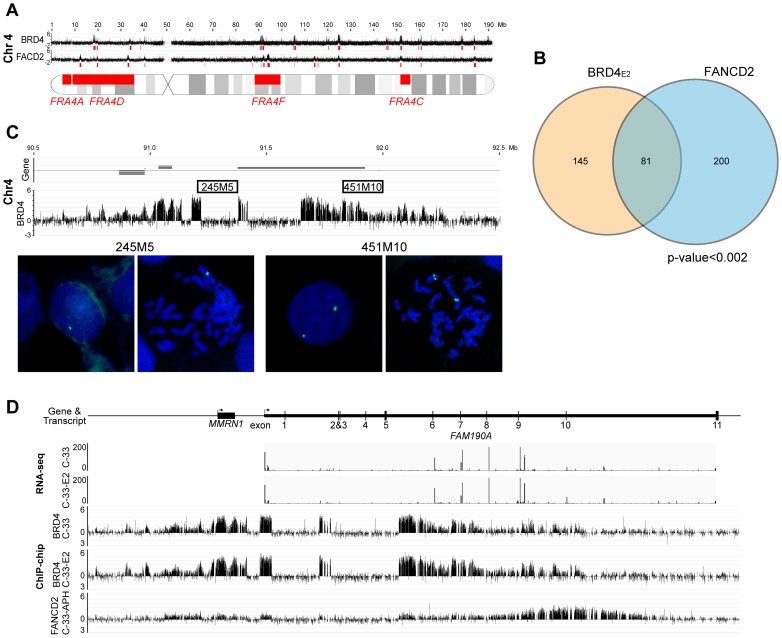
PEB-BLOCs are closely associated with common fragile sites. **A.** An alignment of BRD4 enriched regions (PEB-BLOCs), FANCD2 enriched regions and known common fragile sites on chromosome 4. The red blocks under the signalmap represent computationally defined enriched regions. **B.** Venn diagram showing the overlap in PEB-BLOCs and FANCD2 enriched regions in the entire human genome. The significance of overlap was calculated by the permutation method. **C.** Binding profile of BRD4 in C-33-1E2 cells on PEB-BLOC Chr4-P4, which overlaps the known common fragile site *FRA4F*. FISH analysis with 245M5 or 451M10 probes on interphase and mitotic cells reveal a deletion one allele overlapping the 245M5 probe. **D.** RNAseq analysis of the long *FAM190A* gene in Chr4-P4 PEB-BLOC. Reads of transcripts were aligned with BRD4 ChIP-chip binding data from C-33 and C-33-1E2 cells and FANCD2 binding data from aphidicolin treated C-33 cells.

However, most common fragile sites have been mapped cytogenetically and span large portions of the human genome, making it difficult to statistically correlate with the enriched binding regions. Furthermore, common fragile sites are cell type specific [46] and the majority have been mapped in lymphocytes. To further examine the association of PEB-BLOCs with common fragile sites we mapped aphidicolin-inducible fragile sites in C-33 cells. C-33 cells were treated with aphidicolin to cause mild replication stress and the resulting fragile sites were identified using a novel ChIP-chip method with an antibody to FANCD2, which is involved in replisome surveillance and binds fragile sites [47]–[49]. Approximately 100 strong FANCD2 binding regions were visually identified and are shown aligned with the BRD4 binding profile in Figure S7. Large, enriched FANCD2 binding regions were further defined by computational analysis and are shown as red blocks under the signal map (Figure S7). Figure 6A shows the alignment of PEB-BLOCs, FANCD2 binding sites and known common fragile sites for chromosome 4 and detailed alignments can be found for all strong PEB-BLOCs in Table S1. It is clear that many PEB-BLOCs and FANCD2 binding peaks overlap precisely, others are slightly offset, and some prominent peaks do not overlap. As shown in Figure 6B, a significant subset of FANCD2 enriched binding regions (∼30%) and PEB-BLOCs (∼36%) overlap (P<0.002). Therefore, there is a strong association between PEB-BLOCs and sites of genomic instability. An absolute distance analysis showed that ∼27% PEB-BLOCs and ∼30% FANCD2 enriched binding sites are within 2 Mb of a known common fragile site (Figure S8).

The association between PEB-BLOCs, FANCD2 binding sites and common fragile sites was further examined in three subsets of fragile sites that are most closely related to our biological system. As shown in Table 1 (with details in Table S6) these consisted of: aphidicolin induced fragile sites recently mapped in epithelial cells [50]; fragile sites that have been cloned and therefore are of much higher resolution [51]; and fragile sites that have been mapped in cervical cancer cells [25]. This showed that there was a significant association between these fragile sites and the FANCD2 regions and a near-significant association between these fragile sites and the enriched PEB-BLOC regions.

**Table 1:** Association of common fragile sites with PEB-BLOCs and FANCD2 binding.

Selection of fragile sites*	Enriched regions	# of known common fragile sites	# of overlaps (enriched regions/FRA)	Permutation test (FRA to enriched regions)	
				P-value	Lower tail
***FRA*** **, in epithelial cells** from [50]	BRD4 binding (HPV1 E2)	44	43/19	0.032	TRUE
	FANCD2 binding		55/30	0.002	TRUE
***FRA*** **, cloned** from [51]	BRD4 binding (HPV1 E2)	20	9/6	0.056	TRUE
	FANCD2 binding		16/10	0.030	TRUE
***FRA*** **, cervical cancer** from [25]	BRD4 binding (HPV1 E2)	18	9/6	0.058	TRUE
	FANCD2 binding		16/10	0.032	TRUE

*Sites are listed in Table S6.

*FRA*: fragile sites.

TRUE: when the ‘Lower tail’ is TRUE it indicates that the absolute distances between FRA and enriched binding regions are consistent and small.

### PEB-BLOCs show signs of genomic instability

We noted evidence of deletion in several PEB-BLOCs as there was an abrupt loss of BRD4 signal in certain regions of the BRD4 ChIP-chip binding profiles. We found eight loci showing obvious loss of ChIP signals in PEB-BLOCs and/or FANCD2 binding regions (Figures 6C and S6). Five of these regions are located in the same chromosome bands as known fragile sites, four are in PEB-BLOCs and the others are in non-PEB-BLOC FANCD2 binding regions. To verify these deletions, we performed FISH using two adjacent FISH probes. One probe (245M5) was targeted to the putatively deleted region and the other (451M10) was derived from an adjacent, undeleted BRD4 binding region of the PEB-BLOC. As predicted, the 245M5 probe gave rise to only one FISH signal per cell due to the deletion of this locus on one chromosome (Figure 6C). In contrast, the 451M10 probe showed two clear FISH signals, demonstrating that both chromosomal loci were intact. Because there was an abrupt and complete loss of BRD4 signal in these deleted regions (despite the intact locus on the other chromosome) we can conclude that only the BRD4 bound allele sustained the deletion. Thus, PEB-BLOCs sustain frequent deletions.

This finding is supported by our previous observation that BRD4 is often bound to only one allele of PEB-BLOCs (Figure 5A and 5B). Analysis of the RNAseq signal in these regions confirms that there are no detectable transcripts from the missing exons, reinforcing the hypothesis that the deletion is present in the transcribed allele (Figure 6D). Four of the eight regions shown in Figure S6 also show transcription spanning a deleted allele, supporting this conclusion. Therefore, PEB-BLOCs frequently contain deletions in the transcriptionally active allele.

### Alpha-HPV E1/E2 replication proteins bind to PEB-BLOCs in C-33 cells

**Figure 7:**
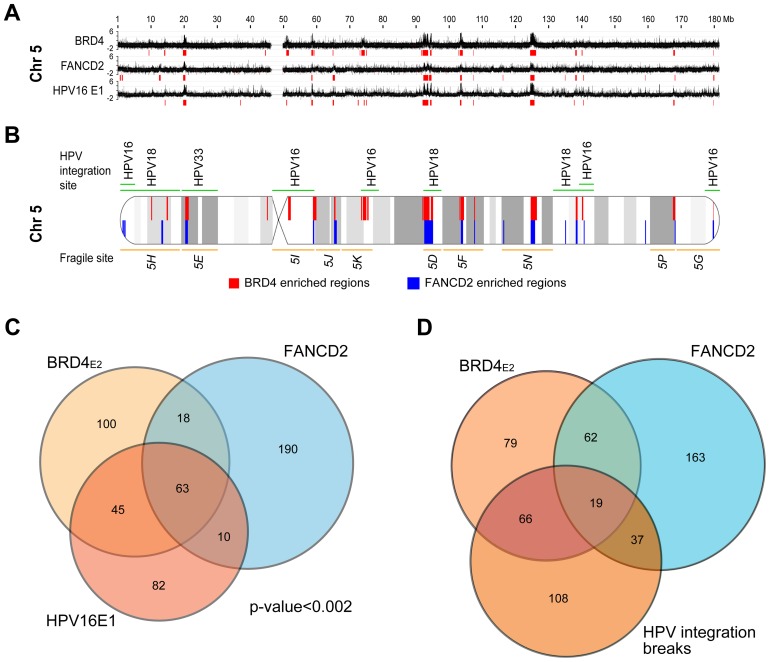
HPV16 E1/E2 binding sites are closely associated with PEB-BLOCs and common fragile sites. **A.** HPV16 E1 and E2 were transiently expressed in C-33 cells. ChIP-chip was performed using an anti-EE (E1) antibody. The Y-axis corresponds to a scaled log_2_-ratio of E1 signal to input signal. Previously obtained BRD4 (C-33-1E2 cells), and FANCD2 (aphidicolin-treated cells) binding profiles are aligned. Enriched regions were computationally defined and are shown in red under the signal map profile. Chromosome 5 is shown here and the entire data is shown in Figure S7. **B.** Diagram of chromosome 5 showing the overlap of BRD4 enriched regions (red), FANCD2 enriched regions (blue), common fragile sites (orange), and HPV integration sites (green) with cytogenetic bands in grey. **C.** A Venn diagram showing the overlap among the enriched BRD4, E1 and FANCD2 binding region regions, as defined by the permutation method. **D.** A Venn diagram showing the overlap among the enriched BRD4 and FANCD2 binding region regions and HPV integration breakpoints as defined by the permutation method. See Table 3 for statistics.

The experiments described above used the E2 protein from HPV1, a virus that causes benign papillomas. The HPV1 E2 protein binds BRD4 with high affinity, but E2 proteins from the Alpha genus have a relatively low affinity for BRD4 and host mitotic chromosomes [9]. Nevertheless, when expressed together with the E1 replication protein both alpha-PV E1 and E2 proteins colocalize in nuclear foci that recruit markers of a DNA damage response (DDR) and recruit BRD4 [11], [29]. Because of the links among E2, BRD4, DDR, replication stress and fragile sites, we questioned whether these nuclear viral replication foci formed at PEB-BLOCs/fragile sites. HPV16 E1 and E2 proteins were transiently expressed in C-33 cells and chromatin was extracted for ChIP-chip analysis. Regions of E1–E2 binding were isolated with an antibody directed against an epitope tag on E1. The resulting E1 binding profile was very similar to that of BRD4 (in the presence of HPV1 E2) and thus to PEB-BLOCS (Figure 7A, 7B and S7). Computation of the E1 enriched regions showed a significant overlap (p<0.002) among the PEB-BLOCs, HPV16 E1 (in the presence of HPV16 E2) and FANCD2 (aphidicolin treated cells) (Figure 7C and Table S5). Therefore, PEB-BLOCs are also targets for alpha-HPV E1/E2 protein complexes and therefore there is a strong link among PEB-BLOCs, fragile sites and viral DNA replication proteins. Highly notable is the fact that HPV genomes are very often integrated close to fragile sites in HPV-associated cancers [25].

### PEB-BLOCs are associated with HPV integration sites

It has been noted for many years that HPVs (and other oncogenic viruses) are often found integrated close to common fragile sites [25], [26], [52]. However, most of the HPV integration sites have been mapped at low resolution, similar to the cytogenetically mapped common fragile sites. To allow for a more detailed analysis, we collated the precise HPV integration sites from several studies [53]–[55] as well as those listed in the DrVIS database [56] (Table S7). The overlap between these sites and the BRD4 and FANCD2 enriched regions is highly significant, as shown in Figure 7D and Table 2. The human genome contains a number of hotspots for HPV integration. For example, chromosomes regions 8q24.21 (the *MYC* locus) and 13q22.1 contain many HPV integration sites [57]; notably these two regions overlap PEB-BLOCs. A recent high resolution study of HPV integration sites in cervical and head and neck cancers demonstrated focal genomic instability; cellular DNA flanking the viral integration site contained amplifications, rearrangements and translocations and concatameric viral DNA was often interspersed with host sequences [58]. Thus, genomic instability continues after the initial integration event.

**Table 2:** Association of HPV integration sites with PEB-BLOCs and FANCD2 binding.

Enriched regions	# of enriched regions	# of HPV integration break points*	# of overlaps between enriched regions and HPV integration breakpoints	Pearson's Chi-squared test		
				p-value	Chi-squared	df
**BRD4 binding (HPV1 E2)**	226	238	85	0.0002438	13.4594	1
**FANCD2 binding**	281	238	56	0.0000631	43.7251	1

*Breakpoints were obtained from [53]–[56]. The regions of analysis were extended to +/−1 Mb from the integration sites. Sites are listed in Table S7.

df: degrees of freedom.

### Papillomavirus replication factories are associated with PEB-BLOCs

Since HPV16 E1 and E2 replication proteins associate with PEB-BLOCs, these are likely sites of viral replication. To verify this we studied the association of HPV genomes with PEB-BLOCs: HPV1, HPV16 and HPV18 genomes were transfected into C-33 cells and the association of viral DNA with specific regions of host chromatin was analyzed by FISH. Transfected viral DNA often gave rise to a single nuclear signal that was closely associated with different PEB-BLOC regions more frequently than control regions (Figure S9).

**Figure 8:**
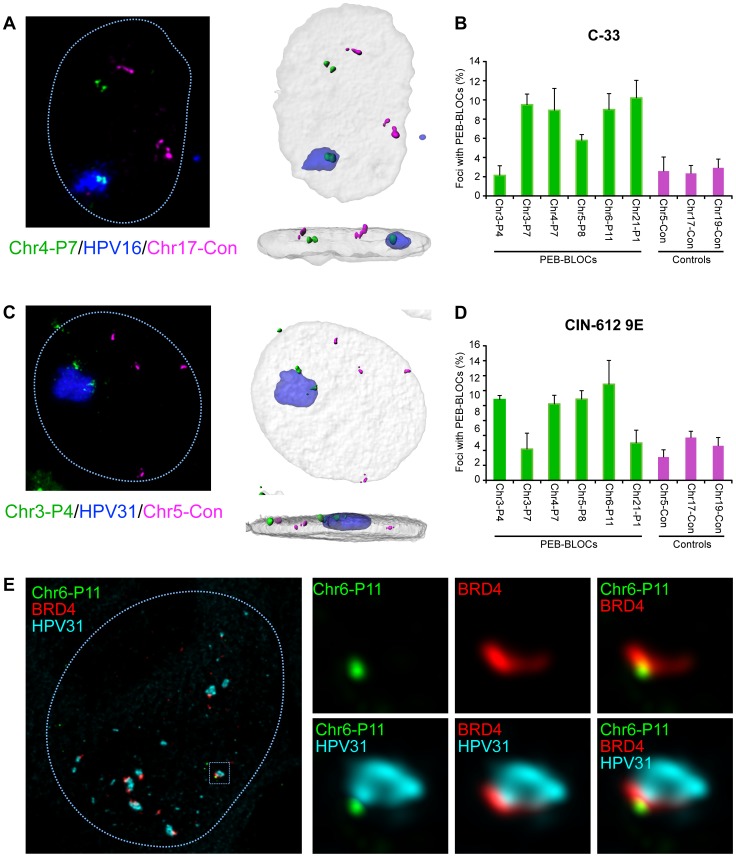
Papillomavirus replication factories are associated with PEB-BLOCs. **A.** C-33 cells containing replicating HPV16 genomes were analyzed by FISH. The representative image shown was probed with HPV16 DNA (blue), Chr4-P7 PEB-BLOC (107E21 BAC clone; green) and a control region (304M13; magenta). 3D image stacks were deconvolved using Huygens Essential software. High resolution surface renderings of all objects were generated by Bitplane Imaris software. **B.** PEB-BLOCs were probed in the cells described in A, using a combination of labeled BAC clones (124A13, 1079D6, 107E21, 126M10, 1062A20 corresponding to PEB-BLOCs Chr2-P6, Chr3-P7, Chr4-P7, Chr5-P8, Chr6-P11), control BAC clones (182E4, 304M13, 568L16 representing negative regions) and HPV16 DNA. Approximately 100 cells were analyzed for colocalization of HPV16, PEB-BLOCs or control regions. Average values and STDEV were calculated for three independent experiments. **C.** Differentiated CIN-612 9E cells were analyzed by FISH. The representative image shown was probed with HPV31 DNA (blue), Chr3-P4 PEB-BLOC (452E16 BAC clone; green) and control region (182E4; magenta). 3D image stacks were deconvolved using Huygens Essential software. High resolution surface renderings of all objects were generated by Bitplane Imaris software. **D.** PEB-BLOCs, HPV31, and negative regions were detected using BAC clones (452E16, 1079D6, 107E21, 126M10, 1062A20, 812E1 corresponding to PEB-BLOCs Chr3-P4, Chr3-P7, Chr4-P7, Chr5-P8, Chr6-P11, Chr21-P1;green), HPV31 DNA (blue), and BAC clones (182E4, 304M13, 568L16 corresponding to negative regions; magenta). >50 HPV31 foci were analyzed for colocalization with PEB-BLOCs or control regions. Average values and STDEV were calculated for three independent experiments. **E.** Differentiated CIN-612 9E cells were analyzed by IF-FISH. BRD4 speckles were identified by immunofluorescence, fixed, and hybridized with a PEB-BLOC specific FISH probe and HPV31 FISH probe. The representative image shown was probed with Chr6-P11 PEB-BLOC (1062A20 BAC clone; green), BRD4 (red), and HPV31 DNA (cyan).

To further explore this association, we isolated C-33 cells containing replicating HPV16 genomes and analyzed the association between the resulting replication foci, PEB-BLOCs and control regions (Figure 8A). Five of the six PEB-BLOCs tested associated with HPV16 replication centers in ∼9% cells while control regions were associated with replication foci in ∼2% cells (Figure 8B). Of importance, the PEB-BLOC allele associated with the replication factory in Figure 8A appears to be late replicating (two, still tightly linked, FISH signals) compared to the “double-dot” pattern observed in the other allele. However, it was difficult to quantitate this observation as the PEB-BLOC signal adjacent to the replication factory was often disrupted and sometimes dispersed throughout the viral DNA. This made it difficult to determine whether it was a singlet or doublet.

When one considers that we are only measuring the interaction of replication factories with one PEB-BLOC at a time (and only a few PEB-BLOCs are likely to be associated with replication foci in any single cell), the observed association is noteworthy. Also of note, some PEB-BLOCs are only associated with HPV replication foci in certain cell lines; for example, Chr3-P4 does not show increased association in C-33 cells, but does in 9E cells (Figure 8). It is possible that the virus replication foci form only at the PEB-BLOC regions with highest affinity for E2 and BRD4.

We carried out a similar analysis in CIN-612 9E cells, which contain large numbers of HPV31 genomes. Large and small viral replication foci can be generated in these cells by differentiation with calcium [27]. Four out of six PEB-BLOCs tested were closely associated with HPV31 replication foci in ∼12% cells, compared to a ∼4% association with control regions (Figure 8C and 8D). In a parallel study, we find that these large foci are frequently ringed with small BRD4 foci [11] that presumably represent additional PEB-BLOCs. In conclusion, replicating HPV genomes are commonly associated with PEB-BLOCs. Figure 8E shows an example of a small replication focus in CIN-612 9E cells stained by immunofluorescence for BRD4, and by FISH for HPV31 DNA and a single PEB-BLOC. As shown, it appears that the replication foci “grow” from the PEB-BLOC foci and BRD4 is localized at the interface between viral and host DNA.

## Discussion

We show that HPV1 E2, and the HPV16 E1/E2 protein complex, bind with BRD4 to common fragile sites in the human genome. Like other persistent viruses that form long-term associations with their host, HPVs are masters at hijacking cellular processes. The E2 proteins interact with BRD4 to regulate viral transcription, and associate with host chromatin to partition the viral genome in dividing cells. We demonstrate that this association is not random, and that the virus has taken advantage of very susceptible regions of the host genome that are prone to replication stress. Both oncogenic and non-oncogenic papillomaviruses induce a DDR and both probably replicate adjacent to these susceptible regions. Most likely, both oncogenic and non-oncogenic HPV types have the propensity to become integrated into unstable regions of the genome on rare occasions. However, only oncogenic HPVs could give the cells a selective growth advantage, in turn further increasing genetic instability, and eventually leading to carcinogenic progression.

The precise role of the BRD4 protein in the HPV lifecycle remains somewhat elusive [19]. BRD4 binds to all PV E2 proteins and regulates viral transcription in an E2-dependent manner. The E2 proteins of viruses such as BPV1 (and HPV1) bind BRD4 with high affinity and link the viral genome to mitotic chromosomes in complex with BRD4, most likely to mediate genome partitioning [4], [59]. However, alpha-PVs (such as HPV16 and HPV31 studied here) bind to BRD4 and chromatin with lower affinity and the role of the E2-BRD4 interaction in replication is enigmatic [10], [11]. In fact, HPV31 genomes encoding an E2 protein that is unable to bind BRD4 in vitro, can replicate persistently, and induce late viral functions, in keratinocytes [60], [61]. One explanation for these findings is that the interaction of E2 and BRD4 is important to establish an efficient infection when limiting amounts of genome are delivered to the nucleus by a viral particle rather than by DNA transfection. Also, the nucleation of viral replication factories at regions of the nucleus highly susceptible to replication stress could be important, but not absolutely required, for efficient viral replication in a natural infection.

While the HPV replication proteins are sufficient to induce a DDR, the viral oncogenes can contribute. E7 induces a DDR by associating with ATM [27] and induces oncogenic replication stress by pushing cells into continual, unscheduled division [62]. This could increase replication stress at fragile sites and potentiate the association with the E2-BRD4-genome complex. Our experiments were carried out in C-33 cancer derived cells and we believe that PEB-BLOCs are very prominent in these cells because they are already very genetically unstable. Thus, under these circumstances E2 and BRD4 bind with high affinity to pre-existing fragile sites without the need for other viral factors to promote replication stress. Normal cells do not show much FANCD2 binding and fragile sites must be induced by replication stress such as that induced by low levels of aphidicolin. In a natural HPV infection, this replication stress could be induced by the viral E7 protein [62].

Common fragile sites are often caused by a paucity of replication origins and/or collisions of transcription and replication machinery in very long genes [32], [63]. Often, fragile sites remain incompletely replicated as cells progress into mitosis. Papillomaviruses amplify their DNA in differentiated cells that are in G2 [64], [65]; hijacking the DDR at this time allows the virus to replicate outside S-phase and without competition from host DNA synthesis. By associating with fragile sites that undergo replication stress at this stage, the virus has to do little but be “in the right place, at the right time”, simply amplifying the DDR response to generate a replication factory. Notably, almost 25 years ago when the correlation between viral integration and fragile sites was first recognized Popescu and DiPaolo predicted that *“It is conceivable that because of their replication pattern at a certain point in the cell cycle fragile sites may be the only replicating regions available for the integration of viral DNA”* [26].

The role of BRD4 in binding to fragile sites has not been completely defined. Previously, chromatin in fragile sites was reported to be hypoacetylated [66], however, we find that the PEB-BLOC regions are highly acetylated and have an “enhancer-like” chromatin signature. It has recently been shown that BRD4 is enriched at super-enhancers that regulate key cell identity genes and tumor drivers [20], [67]. However, despite a common chromatin signature (high H3K4me1 and H3K27ac), PEB-BLOCs are much larger in size than super-enhancers and we do not detect a significant overlap in these elements. Since fragile sites are approaching mitosis with unreplicated regions of DNA, there needs to be a mechanism to keep the chromatin open and accessible to finish replication or repair and to resist the chromosome condensation required for mitosis. BRD4 might maintain an accessible chromatin environment conducive to the processes of DNA damage sensing and repair.

Notably, while BRD4 can preserve chromatin acetylation, decompact chromatin and modulate higher-order chromatin structure [68], a short isoform of BRD4 actually limits the DDR by compacting chromatin to insulate it from ATM signaling [69]. The image shown in Figure 8E is very compatible with the idea that BRD4 is protecting host chromatin from a full-blown viral-mediated DDR. If BRD4 assists in the repair of fragile sites in genetically unstable cells, inactivation of this function could result in a rapid accumulation of catastrophic DNA damage. Normal, genetically stable cells would not depend on this function, and this could help explain the sensitivity of cancer cells to BET inhibitors.

In conclusion, show that the viral E2 and cellular BRD4 proteins associate with fragile regions of the human genome and nucleate replication foci at these sites. This is a resourceful strategy for a virus that uses the host DNA damage response to amplify viral DNA. However, the consequence could be increased accidental integration of viral DNA, which in the case of oncogenic viruses can promote carcinogenesis.

## Materials and Methods

### Plasmids

The pMEP4 expression vectors for FLAG-tagged E2 have previously been described [70]. E2 proteins containing alanine substitutions in residues R37 and I73 were described previously [9]. Standard mutagenesis procedures were used to substitute HPV1 E2 residues R37 and I73 with alanines in pTZ18U-FLAG HPV1 E2. FLAG-HPV1 E2 (R37A/I73A) was subcloned into the Asp718 and blunted NheI sites of pMEP4. The FLAG-HA tag was introduced into the HindIII and blunted NotI sites of pMEP4 to generate the control plasmid, pMEP-fh. Plasmids expressing HPV16 E1 and E2 proteins have been described previously [29]. RPCI-11 BAC clones were purchased from Empire genomics (Table S10). HPV1, HPV16, HPV18 and HPV31 genomes have been described previously [24], [71]–[73] and sequences can be found at http://pave.niaid.nih.gov [74].

### Antibodies

All antibodies are described in Table S8.

### BET Inhibitors

GSK525762^+^ or the inactive enantiomer GSK525762^−^ were synthesized as described previously [11], following the methods described [38].

### Cells

C-33 cells [75] were cultured in DMEM, 10% FBS, 100 U/ml penicillin, and 100 µg/ml streptomycin. The HPV31 positive cell line, CIN-612 9E cells [76] were obtained from Lou Laimins (Northwestern University, Chicago, Illinois, USA) and was grown on irradiated 3T3-J2 feeder cells in F medium (3∶1 [v/v] F-12 [Ham]-DMEM, 5% FBS, 0.4 µg/ml hydrocortisone, 5 µg/ml insulin, 8.4 ng/ml cholera toxin, 10 ng/ml EGF, 24 µg/ml adenine, 100 U/ml penicillin, and 100 µg/ml streptomycin).

### Establishment of E2 expressing cells and transient expression of HPV16 E1 and E2

Inducible E2 expressing cell lines were generated in an HPV-negative cervical carcinoma derived cell line, C-33 by transfecting with the pMEP4-E2 expression plasmids, using Fugene (Roche). Cells containing the pMEP episomal plasmids were selected with 80 µg/ml of hygromycin B (Roche). Drug-resistant colonies were pooled after 2 weeks. E2 protein expression was induced with CdSO_4_ for 4 h before harvest and the levels of E2 proteins were titrated and adjusted by differential CdSO_4_ concentration to ensure that binding to the identified chromatin regions increased in an E2-dependent and specific fashion. For transient HPV16 E1/E2 expression, C-33 cells were cotransfected with pMEP9/EE-HPV16 E1 and pMEP4/FLAG-HPV16 E2. E2 expression was induced with 3 µM CdSO_4_ induction for 4 h before harvest at 24 h post-transfection.

### Differentiation of CIN-612 9E cells with calcium

CIN-612 9E cells were differentiated with calcium, essentially as described previously [27]. Feeders and CIN-612 9E cells were seeded as described above. When 90% confluent, the medium was changed to Lonza Growth medium (KBM plus supplement media). Twenty four h later, the medium was changed to Differentiation medium (Lonza KBM/1.5 mM CaCl_2_/no supplements). Cells were cultured for the times indicted before harvest or fixation.

### Induction of common fragile sites

C-33 cells were treated with 0.2 µM aphidicolin for 24 h before harvesting for ChIP-chip experiments described in other sections.

### Chromatin immunoprecipitation-chip

ChIP experiments were performed as previously described [34]. For mitotic cells, C-33 cells were blocked by 2 mM thymidine overnight and released into medium without thymidine for 9 h. Four hours before harvesting, E2 expression was induced with 3 µM CdSO_4_ and mitotic cells were collected by mitotic shake off at which point cells were fixed in formaldehyde. For conventional ChIP assay, 0.5 mg of chromatin prepared from asynchronous or mitotic cells was incubated overnight with a specific antibody and collected with Dynabeads conjugated to Protein G (Invitrogen). For ChIP-chip analysis, 2 mg of chromatin was incubated overnight with a specific antibody prebound to Dynabeads conjugated to Protein G. Further processing for ChIP-chip was as described by Jang et al. [34]. DNA isolated from immunoprecipitated chromatin was amplified using the whole genome amplification system (WGA, Sigma). Two HG18 build whole genome arrays were used. C-33-1E2 amplified DNA was labeled and hybridized to the 385K Whole-Genome Tiling Array or the 2.1M Whole-Genome Tiling Array by NimbleGen. E2 binding signals on the arrays for ChIP DNA were normalized to the input signals for total DNA. The ratios were plotted against genomic position to identify regions where increased signal is observed relative to the control sample. All datasets are available at GEO: GSE52312.

### Real-time quantitative PCR

Real-time Q-PCR was performed using the ABI Prism 7900HT Sequence Detector (Applied Biosystems) and SYBR Green PCR master mix (Applied Biosystems). An aliquot of ChIP DNA was analyzed with 12.5 µl of SYBR Green PCR master mix and 0.3 µM each oligonucleotide primer in total volume of 25 µl. In each run, a four-fold dilution series of pooled input chromatin DNA was used to generate a standard curve of threshold cycle (Ct) versus log of quantity. PCR was performed at 95°C for 15 min, followed by 40 cycles of denaturation at 95°C for 10 sec and annealing and extension at 60°C for 60 sec. The specificity of each primer pair was determined by dissociation curve analysis. The data were analyzed with SDS 2.1 software (Applied Biosystems). The primers used are listed in Table S9.

### Indirect immunofluorescence

Cells were arrested in G1/S phase by culture in 2 mM thymidine overnight, washed to release, and grown for 9 h in the absence of thymidine to select for cells in mitosis. The metallothioneine promoter was induced by the addition of 3 µM CdSO_4_ for 4 h. Cells were fixed at room temperature in 4% paraformaldehyde (PFA) in PBS for 20 minutes, blocked and stained with mouse monoclonal anti-FLAG M2 antibody and FITC or Alexa 488 anti-mouse antibody; various primary rabbit antibodies and Texas Red or Alexa 596 anti-rabbit antibody. Cellular DNA was stained with DAPI. Images were collected using a Leica TCS-SP5 laser scanning confocal imaging system.

### Downregulation of BRD4 expression by siRNA treatment

Cells were seeded at a density of 1×10^6^ cells per 10 cm dish, incubated for 24 h, and transfected with 750 ng of siRNA (Table S11) using 40 µl of HiPerFect (Qiagen). Cells were incubated for three days at which point E2 expression was induced by 3 µM CdSO_4_ for 4 h. The efficiency of BRD4 downregulation was verified by immunoblot analysis using specific antibodies for the target proteins. siRNA treated cells were fixed for immunofluorescence using specific antibodies, as described above.

### Fluorescence in situ hybridization (FISH)

Cells were cultured on coverslips or glass slides. 9E cells were differentiated for 5 days in the KBM media with 1.5 mM CaCl_2_. The cells were fixed three times with cold methanol∶acetic acid (3∶1) for 15 mins. For chromosome spreads, C-33 cells were prepared as described for indirect immunofluorescence, treated with 0.1 mg/ml of Colcemid Karyomax (Invitrogen) for 90 mins, and collected by mitotic shake-off. Cells were resuspended in 10 ml of hypotonic buffer (0.075 M KCl) and incubated at 37°C for 20 mins. After pelleting, the cells were resuspended and fixed three times in 10 ml of cold methanol∶acetic acid (3∶1) for 15 mins. The fixed cells were resuspended in 0.5 ml methanol∶acetic acid, applied onto glass slide by dropping, and dried for O/N. The cells were treated with RNace-it cocktail for 1 h, dehydrated with 70%, 85%, and 100% ethanol, and dried for several hours. For combined immunofluorescence-FISH analysis, mitotic cells were collected as described above and treated with H1 buffer (10 mM Tris, pH 7.4, 10 mM NaCl, 5 mM MgCl_2_) for 15 mins and H2 buffer (0.25× PBS) for 15 mins. Cells were centrifuged at spun at 1500 rpm for 10 mins in a Cytocentrifuge 7620 (Wescor) and fixed at room temperature in 4% PFA/PBS for 20 minutes. After immunofluorescent detection as described above, cells were treated with methanol∶acetic acid (3∶1) for 10 min, 2% paraformaldehyde for 1 min, before dehydration through a series of 70%, 90%, and 100% ethanol.

FISH probes were prepared using ULysis nucleic acid labeling kit (Molecular Probes), purified through Illustra ProbeQuant G-50 micro column (GE Healthcare), and resuspended in TE containing 0.3 µg/µl of Cot-1 DNA. For hybridization, 2 µl 5-fluorescein-labeled BAC probe (Empire Genomics) or 50 ng ULysis FISH probe was mixed with 8 µl FISH hybridization buffer (Empire Genomics), applied to the slide, covered with coverslip, and sealed with rubber cement. The cells and probes were denatured at 75°C for 5 minutes and incubated overnight at 42°C. Cells were washed in 1× phosphate-buffered detergent (Qbiogene) for 5 min at room temperature, 1× wash buffer (0.5× SSC, 0.1% SDS) for 5 min at 65°C, and 1× phosphate-buffered detergent (Qbiogene) for 5 min at room temperature. Cellular DNA was stained with DAPI. Images were collected using a Leica TCS-SP5 laser scanning confocal imaging system. Images were processed using Leica AS Lite software, or Bitplane Imaris software (Zurich, Switzerland) or deconvolved with Huygens Essential software (Scientific Volume Imaging B.V., VB Hilversum, Netherlands), where indicated.

### RNA-seq

C-33 cells were seeded at a density of 1×10^6^ cells per 10 cm dishes and grown for 2 days. E2 expression was induced by the addition of 3 µM CdSO_4_ for 4 h. Total RNA was purified using RNeasy (Qiagen), and analyzed for integrity using the Agilent RNA 6000 nano kit on 2100 Bioanalyzer (Agilent). Both polyadenylated and non-polyadenylated (after rDNA subtraction) RNA was sequenced. Two different libraries were constructed for each sample. For one library, total RNA was purified by poly A selection following manufacturer's instructions. For the second library, 1.5 µg total RNA was rRNA depleted using Ribo-Zero (Epicentre, Madison, WI), followed by library generation using the Illumina TruSeq RNA protocol, beginning at the fragmentation step. Libraries were sequenced on an Illumina GAIIx. The adapters were trimmed from raw sequences and low quality reads were filtered out. Processed reads were mapped to human genome assembly hg19 using Tophat and differentially expressed gene analysis was performed using Cufflinks [77]. Data was visualized using the Integrative Genomics Viewer (Broad Institute). The dataset can be accessed at GEO: GSE52367.

### Introduction and detection of recircularized papillomavirus genomes in C-33 cells

HPV genomes were removed from the plasmid vector by restriction digestion and religated as described [78]. C-33 cells expressing either HPV1, HPV16, or HPV18 E2 proteins were transfected using FuGene 6 with the corresponding recircularized HPV genome and incubated for 24 h. E2 expression was induced with 3 µM CdSO_4_ for 4 h and the cells were prepared for FISH experiments as described above. PEB-BLOCs were detected using 5-fluorescein or Alexa 488 labeled probes, produced from BAC clones by Empire Genomics (Table S10), and the HPV genomes were detected using HPV DNA, purified from vector sequences by PCR amplification and labeled by an Alexa 594 ULysis labeling kit (Molecular Probes).

### Bioinformatics and statistics

#### Identification of ChiP-enriched regions

NimbleGen ChIP-chip microarray raw data files were processed with the R/Bioconductor package Ringo [79], [80]. The probe intensity was calculated as the log2 ratio of cy5/cy3. Tukey's biweight normalization was performed to correct systematic dye and labeling biases and probe intensity was smoothed using a 1000 bp width to reduce systematic and stochastic noise along chromosomes. The smoothed intensity was fitted by a mixture model to determine the enrichment threshold. ChIP-enriched regions were defined as regions having at least 10 probes whose smoothed intensity was larger than the enriched threshold, and the distance between these probes was less than 5000 bp. If the distance between two identified enriched regions was less than 50 kbp, these two regions were further combined into a larger enriched region. Enriched regions of less than 50 kbp were also filtered out from further analysis.

#### Identification of overlapped regions

If two genomic regions Ri and Rj satisfy the following formula, length (Ri ∩ Rj)≥0.5 * min(length(Ri), length(Rj)), they were defined as overlapped.

#### Chi-squared contingency table test

A chi-squared contingency table test was performed to compare two genomic regions based on the two by two table shown in Table 3, where A represents the number of overlapped regions between E1 and E2; B represents the number of overlapped regions between E1 and NE2; C represents the number of overlapped regions between NE1 and E2; and D represents the number of overlapped regions between NE1 and NE2.

**Table 3:** Chi-squared contingency table test.

	ChiP-Enriched region from experiment 2 (E2)	Non-ChiP-Enriched region from experiment 2 (NE2)
ChiP-Enriched region from experiment 1 (E1)	A	B
Non-ChiP-Enriched region from experiment 1 (NE1)	C	D

A chi-squared contingency table test was performed to compare two genomic regions where A represents the number of overlapped regions between E1 and E2; B represents the number of overlapped regions between E1 and NE2; C represents the number of overlapped regions between NE1 and E2; and D represents the number of overlapped regions between NE1 and NE2.

#### Absolute distance test

Absolute distance test was performed to determine whether the nearest distance between ChiP-enriched regions and reference regions are closer than the nearest distance between randomly distributed regions and reference regions using software package GenometriCorr [81].

## Supporting Information

Figure S1 **Examples of E2 mitotic binding regions and comparison of E2 binding to broad persistent regions vs promoter regions.** **A.** C33 control (pM4) or C33-1E2 cells were treated with different concentration of CdSO_4_ for 4 h. E2 protein levels were monitored by immunoblotting with an anti-FLAG antibody and alpha-tubulin levels were monitored as a loading control. **B.** Different levels of E2 protein were analyzed for binding to persistent chromatin binding sites by ChIP. E2-bound DNA fragments were purified by IP using anti-FLAG M2 antibody or control IgG and detected using real-time PCR with specific primers for the persistent binding sites. **C.** Representative examples of mitotic HPV1 E2 binding regions, located at chr4:151,160,500–151,780,500 and chr5:123,942,100–125,482,100 annotated with known genes. PCR primers used in B–D are indicated by black arrows for E2 binding regions (chr5: 124,082,100; 124,240,100; 124,455,100; 124,624,100; 124,746,100) and a red arrow for the E2-negative binding region (chr5:124,145,000). **D–F.** BRD4 binding to mitotic chromatin in the presence or absence of HPV1 E2 expression to several regions within chr5:123,942,100–125,482,100 and to promoter regions (BRD2, CCD1, SALL4, TUBB) previously shown to bind E2 and BRD4 in asynchronous cells (Jang et al., 2009). Cells were synchronized by thymidine block and released to enrich for mitotic cells. Four hours before harvest, E2 expression was induced with 3 µM CdSO4. Mitotic cells were collected by shake-off and fixed in 1% formaldehyde. Chromatin was purified by IP using IgG, anti-FLAG M2, and anti-BRD4 antibodies and detected using Q-PCR with the primers described in Table S9. PCR primers used were from E2 binding regions (chr5: 124,082,100; 124,240,100; 124,455,100; 124,624,100; 124,746,100) and an E2-negative binding region (chr5:124,145,000). Average values and STDEV are shown. TSS: transcriptional start site. **G.** A representative comparison of E2 and BRD4 binding to broad persistent regions (as detected by whole genome arrays in mitotic C33 cells expressing HPV1 E2) and promoter regions (as detected in asynchronous cells C33 cells expressing BPV1 E2 by promoter arrays). The latter data was published previously in Jang et al., 2009. The region shown is from chromosome 4: l23,500,000–126,500,000.
(PDF)

Figure S2 **Profile of BRD4 binding on chromosomes in C-33 cells.** C-33 cells containing either a tag only vector (pMEP4 fh) or pMEP4-HPV1 E2 were treated with 1 µM CdSO_4_ for 4 h and fixed with 1% formaldehyde. Chromatin DNA samples were prepared by ChIP using the anti-BRD4 antibody, amplified with the whole genome amplification method and characterized by hybridization to 2.1M human whole genome arrays by NimbleGen. The BRD4 binding signals in C-33 cells were obtained and aligned for entire chromosomes using the SignalMap program. Enriched regions of BRD4 binding were defined computationally and are shown in red, listed in Table S3, and labeled Bcon (BRD4 binding in control cells) and BE2 (Brd4 binding in E2 expressing cells). Some of the BRD4 binding regions were detectable in control cells and HPV1 E2 expressing cells. Other BRD4 binding regions were undetectable in control cells and were significantly increased by HPV1 E2 expression. The chromosomal nucleotide positions are shown along the top. The Y-axis corresponds to a scaled log2-ratio of E2 signal to input signal.
(PDF)

Figure S3 **Persistent E2 binding regions have distinctive patterns of histone modification.** Chromatin samples from mitotic HPV1 E2-expressing C-33 cells were subjected to ChIP, as shown in Figure 3F, and further analyzed with specific antibodies against histone H3, H3K9ac, H3K14ac, H3K18ac, H3K23ac, H3K27ac, H3K9ac/K14ac, H3K9ac/K18ac, H4K5ac, H4K12ac, H4K5ac/K8ac/K12ac/K16ac, and H3K36me3. ChIP DNA was analyzed by quantitative real-time PCR with primer sets for the specific E2 binding regions (see Table S3). ChIP signals were expressed as percentage of chromatin DNA immunoprecipitated from the input amount of chromatin. Average values and STDEV were calculated for 3 independent experiments on 4 non-E2 binding regions, 4 transcriptionally active promoters, and 6 persistent binding sites.
(PDF)

Figure S4 **Co-localization of E2 and BRD4 and modified histones in mitotic cells.** Immunofluorescence of mitotic C-33 cells expressing HPV1 E2 was performed with specific antibodies against HPV1 E2 (FLAG), BRD4, H3K56ac, H4K8ac, H3K4me1, H3K4me2, and H3K4me3. E2 protein is shown in green and BRD4 protein or the modified histones are shown in red. Cells were counterstained by DAPI (blue).
(PDF)

Figure S5 **BRD4 colocalizes with PEB-BLOCs in interphase nuclei.** C-33-1E2 cells were stained by immunofluorescence with an anti-BRD4 antibody followed by fixation. The cells were subsequently hybridized with specific FISH probes for 12 individual PEB-BLOCs. The chromosomal position of each PEB-BLOC and number of the FISH probe is shown above each panel. Image stacks were deconvolved using Huygens Essential software. Signals from IF and FISH were detected and are shown in red (BRD4) and green (FISH). The nuclei (stained with DAPI; not shown) are outlined in blue.
(PDF)

Figure S6 **Deletions are common in PEB-BLOCs.** A selection of eight regions (4 PEB-BLOCs in the top row and 4 FANCD2 binding sites in the bottom row) containing deletions in C-33 cells. The BRD4 in C-33 cells (BRD4con), C-33 cells expressing E2 (BRD4_E2_) and FANCD2 binding signals are shown. The deletions are underscored with a red line.
(PDF)

Figure S7 **FANCD2 binding in aphidicolin treated C-33 cells and E1 binding in HPV16 E1/E2 expressing cells.** C-33 cells were treated with 0.2 µM aphidicolin for 24 h to cause mild replication stress before chromatin isolation. Alternatively, C-33 cells were transiently transfected with pMEP9-HPV16 E1 and pMEP4-HPV16 E2 for 24 h and treated with 3 µM CdSO_4_ for 4 h before harvesting. ChIP was performed using an anti-FANCD2 antibody or anti-EE antibody, respectively. ChIP-chip was performed as described for Materials and Methods. The chromosomal nucleotide positions are shown along the top. The Y-axis corresponds to a scaled log_2_-ratio of binding signal for FANCD2 or HPV16 E1 to input signal. The binding profiles are aligned with previously obtained BRD4 signals in the presence of E2 expression (Figure S2) for the entire set of human chromosomes. Enriched regions of BRD4 binding, FANCD2 binding and HPV16 E1 binding were defined computationally and are shown in red, and listed in Table S5.
(PDF)

Figure S8 **Histograms of nearest distance for common fragile sites and enriched regions of BRD4 and FANCD2 binding.** Absolute distance tests were performed to determine whether the distance between enriched regions of BRD4 and FANCD2 binding are closer to common fragile sites than to randomly distributed regions. The common fragile sites are those mapped in epithelial cells by [50] and are listed in Table S6. **A.** Histogram of nearest distance for PEB-BLOCs and cFRA. **B.** Histogram of nearest distance for FANCD2 enriched regions and cFRA.
(PDF)

Figure S9 **Papillomavirus virus genomes are often associated with PEB-BLOCs.** C-33 cells were transfected with HPV1, HPV16 or HPV18 viral genomes, which were detected by FISH five days post-transfection. PEB-BLOCs were detected using BAC clones (1062A20 and 124A13 in green) corresponding to PEB-BLOCs (Chr2-P6 and Chr6-P11) and viral DNA is shown (red). A representative image is shown for each HPV with the specific PEB-BLOC region indicated. Only a small percentage of replication foci show association with individual PEB-BLOC probes, but this is consistent with the hypothesis that each focus associates with only a few PEB-BLOCs.
(PDF)

Table S1 **Characteristics of PEB-BLOCs.**
(PDF)

Table S2 **List of E2, BRD4 binding sites used for analysis in** **Figure 1A** **and** **2A** **.**
(XLSX)

Table S3 **List of BRD4 binding sites used for analysis in Figure S2.**
(XLSX)

Table S4 **List of E2, BRD4, Histone binding sites used for analysis in** **Figure 4B** **.**
(XLSX)

Table S5 **List of BRD4, FancD2, 16E1 binding sites used for analysis in Figure S7.**
(XLSX)

Table S6 **List of specialized FRA site coordinates used for analysis in** **Table 1** **.**
(XLSX)

Table S7 **List of HPV breakpoints used for analysis in** **Table 3** **.**
(XLSX)

Table S8 **List of antibodies.**
(PDF)

Table S9 **List of Q-PCR primers.**
(PDF)

Table S10 **List of Bac clones.**
(PDF)

Table S11 **List of siRNAs.**
(PDF)
