# Supplementary material for: Papillomavirus Genomes Associate with BRD4 to Replicate at Fragile Sites in the Host Genome
Source: PLoS Pathog. 2014 May 15;10(5):e1004117. doi: 10.1371/journal.ppat.1004117 (PMC4022725; doi:10.1371/journal.ppat.1004117)
Supplement: Figure S1 — Examples of E2 mitotic binding regions and comparison of E2 binding to broad persistent regions vs promoter regions. A. C33 control (pM4) or C33-1E2 cells were treated with different concentration of CdSO4 for 4 h. E2 protein levels were monitored by immunoblotting with an anti-FLAG antibody and alpha-tubulin levels were monitored as a loading control. B. Different levels of E2 protein were analyzed for binding to persistent chromatin binding sites by ChIP. E2-bound DNA fragments were purified by IP using anti-FLAG M2 antibody or control IgG and detected using real-time PCR with specific primers for the persistent binding sites. C. Representative examples of mitotic HPV1 E2 binding regions, located at chr4:151,160,500–151,780,500 and chr5:123,942,100–125,482,100 annotated with known genes. PCR primers used in B–D are indicated by black arrows for E2 binding regions (chr5: 124,082,100; 124,240,100; 124,455,100; 124,624,100; 124,746,100) and a red arrow for the E2-negative binding region (chr5:124,145,000). D–F. BRD4 binding to mitotic chromatin in the presence or absence of HPV1 E2 expression to several regions within chr5:123,942,100–125,482,100 and to promoter regions (BRD2, CCD1, SALL4, TUBB) previously shown to bind E2 and BRD4 in asynchronous cells (Jang et al., 2009). Cells were synchronized by thymidine block and released to enrich for mitotic cells. Four hours before harvest, E2 expression was induced with 3 µM CdSO4. Mitotic cells were collected by shake-off and fixed in 1% formaldehyde. Chromatin was purified by IP using IgG, anti-FLAG M2, and anti-BRD4 antibodies and detected using Q-PCR with the primers described in Table S9. PCR primers used were from E2 binding regions (chr5: 124,082,100; 124,240,100; 124,455,100; 124,624,100; 124,746,100) and an E2-negative binding region (chr5:124,145,000). Average values and STDEV are shown. TSS: transcriptional start site. G. A representative comparison of E2 and BRD4 binding to broad persistent regions (as detected [file ppat.1004117.s001.pdf]

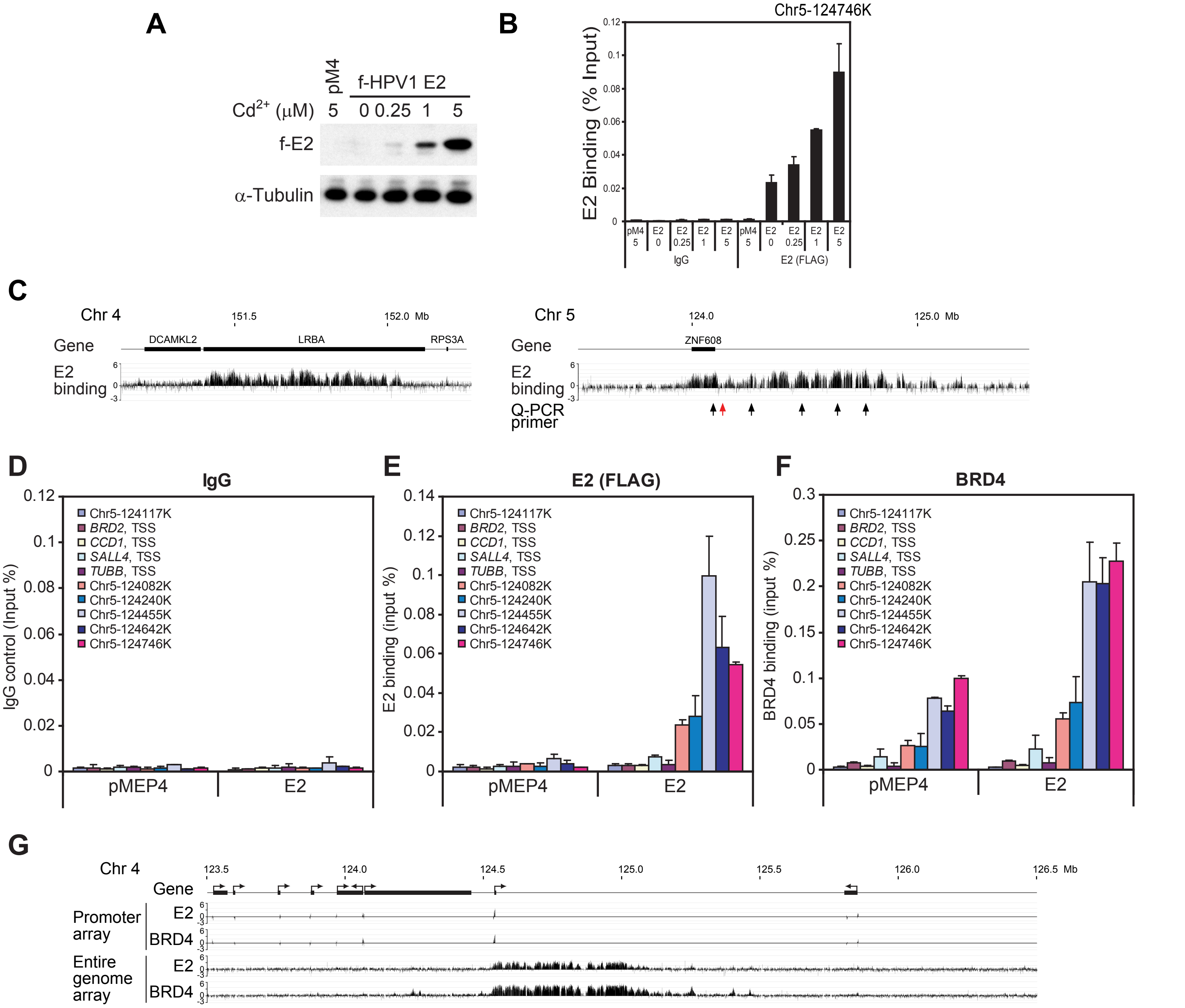

Figure S1. Examples of E2 Mitotic Binding Regions and Comparison of E2 Binding to Broad Persistent Regions vs Promoter Regions

A. C33 control (pM4) or C33-1E2 cells were treated with different concentration of $CdSO_4$ for 4 h. E2 protein levels were monitored by immunoblotting with an anti-FLAG antibody and alpha-tubulin levels were monitored as a loading control.

B. Different levels of E2 protein were analyzed for binding to persistent chromatin binding sites by ChIP. E2-bound DNA fragments were purified by IP using anti-FLAG M2 antibody or control IgG and detected using real-time PCR with specific primers for the persistent binding sites.

C. Representative examples of mitotic HPV1 E2 binding regions, located at chr4:151,160,500- 151,780,500 and chr5:123,942,100-125,482,100 annotated with known genes. PCR primers used in B-D are indicated by black arrows for E2 binding regions (chr5: 124,082,100; 124,240,100; 124,455,100; 124,624,100; 124,746,100) and a red arrow for the E2-negative binding region (chr5:124,145,000).

D-F. BRD4 binding to mitotic chromatin in the presence or absence of HPV1 E2 expression to several regions within chr5:123,942,100-125,482,100 and to promoter regions (*BRD2, CCD1, SALL4, TUBB*) previously shown to bind E2 and BRD4 in asynchronous cells (Jang et al., 2009). Cells were synchronized by thymidine block and released to enrich for mitotic cells. Four hours before harvest, E2 expression was induced with 3 μM $CdSO_4$. Mitotic cells were collected by shake-off and fixed in 1% formaldehyde. Chromatin was purified by IP using IgG, anti-FLAG M2, and anti-BRD4 antibodies and detected using Q-PCR with the primers described in Table S9. PCR primers used were from E2 binding regions (chr5: 124,082,100; 124,240,100; 124,455,100; 124,624,100; 124,746,100) and an E2-negative binding region (chr5:124,145,000). Average values and STDEV are shown. TSS: transcriptional start site.

G. A representative comparison of E2 and BRD4 binding to broad persistent regions (as detected by whole genome arrays in mitotic C33 cells expressing HPV1 E2) and promoter regions (as detected in asynchronous cells C33 cells expressing BPV1 E2 by promoter arrays). The latter data was published previously in Jang et al., 2009. The region shown is from chromosome 4: l23,500,000-126,500,000.
