# Supplementary material for: Papillomavirus Genomes Associate with BRD4 to Replicate at Fragile Sites in the Host Genome
Source: PLoS Pathog. 2014 May 15;10(5):e1004117. doi: 10.1371/journal.ppat.1004117 (PMC4022725; doi:10.1371/journal.ppat.1004117)
Supplement: Figure S2 — Profile of BRD4 binding on chromosomes in C-33 cells. C-33 cells containing either a tag only vector (pMEP4 fh) or pMEP4-HPV1 E2 were treated with 1 µM CdSO4 for 4 h and fixed with 1% formaldehyde. Chromatin DNA samples were prepared by ChIP using the anti-BRD4 antibody, amplified with the whole genome amplification method and characterized by hybridization to 2.1M human whole genome arrays by NimbleGen. The BRD4 binding signals in C-33 cells were obtained and aligned for entire chromosomes using the SignalMap program. Enriched regions of BRD4 binding were defined computationally and are shown in red, listed in Table S3, and labeled Bcon (BRD4 binding in control cells) and BE2 (Brd4 binding in E2 expressing cells). Some of the BRD4 binding regions were detectable in control cells and HPV1 E2 expressing cells. Other BRD4 binding regions were undetectable in control cells and were significantly increased by HPV1 E2 expression. The chromosomal nucleotide positions are shown along the top. The Y-axis corresponds to a scaled log2-ratio of E2 signal to input signal. (PDF) [file ppat.1004117.s002.pdf]

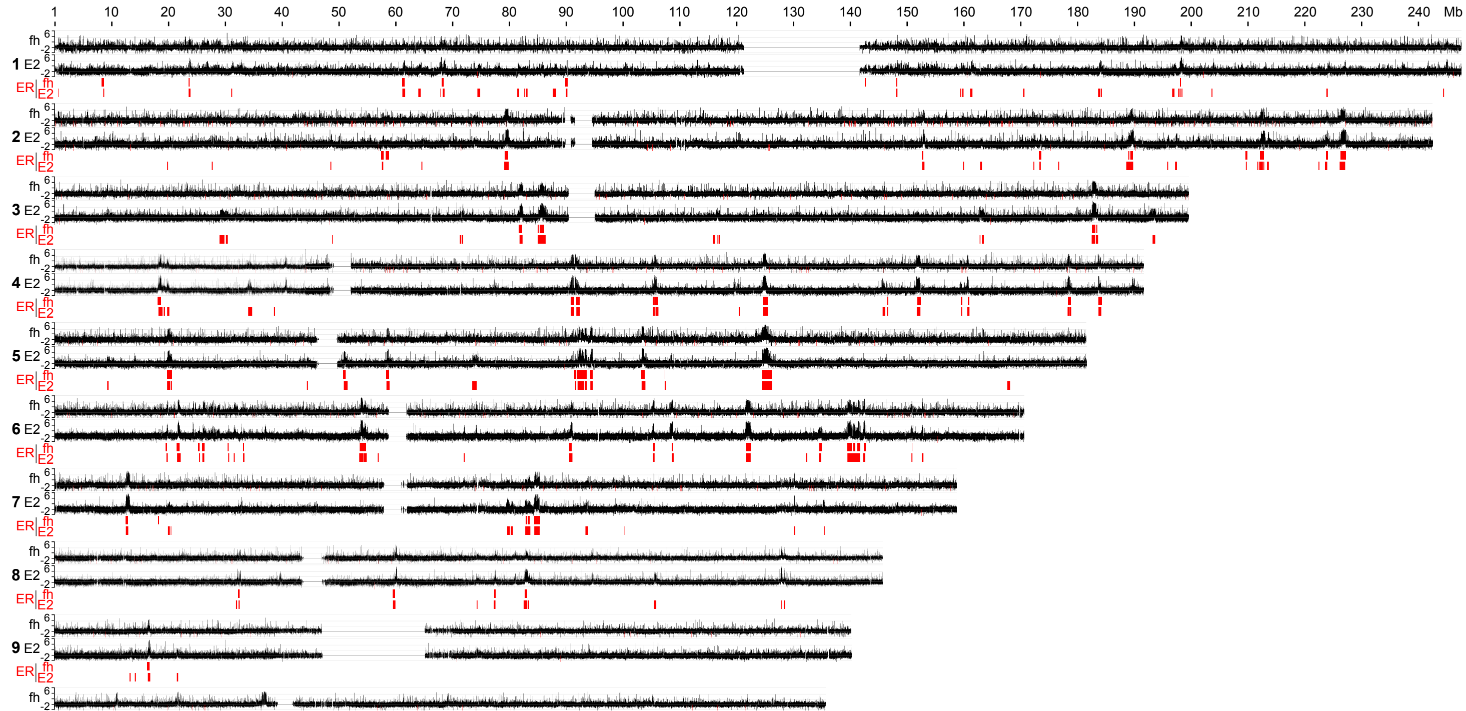

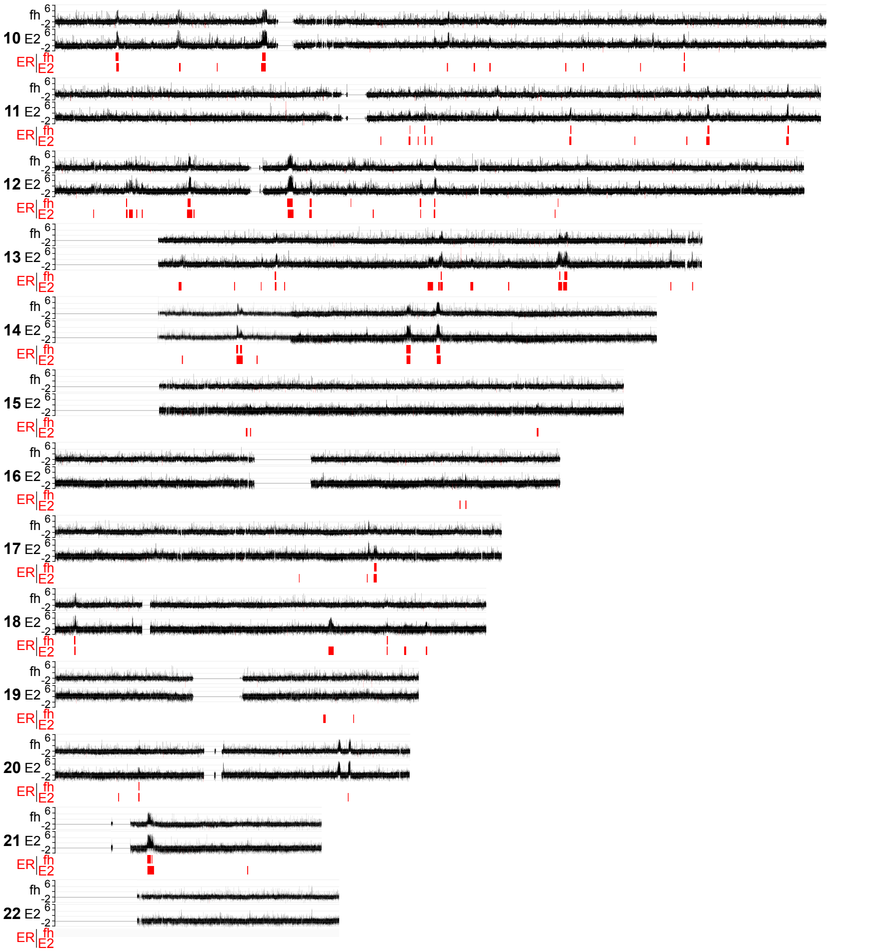

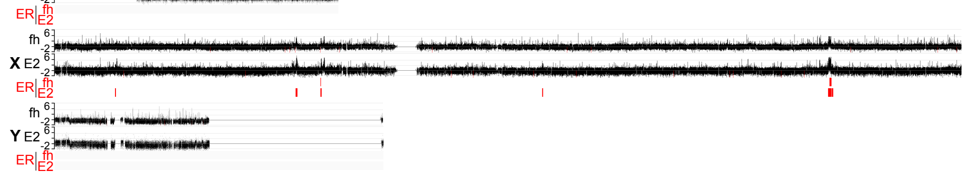

Figure S2. Profile of BRD4 binding on chromosomes in C-33 cells

C-33 cells containing either a tag only vector (pMEP4 fh) or pMEP4-HPV1 E2 were treated with 1 μM $CdSO_4$ for 4 h and fixed with 1% formaldehyde. Chromatin DNA samples were prepared by ChIP using the anti-BRD4 antibody, amplified with the whole genome amplification method and characterized by hybridization to 2.1M human whole genome arrays by NimbleGen. The BRD4 binding signals in C-33 cells were obtained and aligned for entire chromosomes using the SignalMap program. Enriched regions of BRD4 binding were defined computationally and are shown in red, listed in Table S3, and labeled B_{con} (BRD4 binding in control cells) and B_{E2} (Brd4 binding in E2 expressing cells). Some of the BRD4 binding regions were detectable in control cells and HPV1 E2 expressing cells. Other BRD4 binding regions were undetectable in control cells and were significantly increased by HPV1 E2 expression. The chromosomal nucleotide positions are shown along the top. The Y-axis corresponds to a scaled log2-ratio of E2 signal to input signal.
