# Supplementary material for: Papillomavirus Genomes Associate with BRD4 to Replicate at Fragile Sites in the Host Genome
Source: PLoS Pathog. 2014 May 15;10(5):e1004117. doi: 10.1371/journal.ppat.1004117 (PMC4022725; doi:10.1371/journal.ppat.1004117)
Supplement: Figure S3 — Persistent E2 binding regions have distinctive patterns of histone modification. Chromatin samples from mitotic HPV1 E2-expressing C-33 cells were subjected to ChIP, as shown in Figure 3F, and further analyzed with specific antibodies against histone H3, H3K9ac, H3K14ac, H3K18ac, H3K23ac, H3K27ac, H3K9ac/K14ac, H3K9ac/K18ac, H4K5ac, H4K12ac, H4K5ac/K8ac/K12ac/K16ac, and H3K36me3. ChIP DNA was analyzed by quantitative real-time PCR with primer sets for the specific E2 binding regions (see Table S3). ChIP signals were expressed as percentage of chromatin DNA immunoprecipitated from the input amount of chromatin. Average values and STDEV were calculated for 3 independent experiments on 4 non-E2 binding regions, 4 transcriptionally active promoters, and 6 persistent binding sites. (PDF) [file ppat.1004117.s003.pdf]

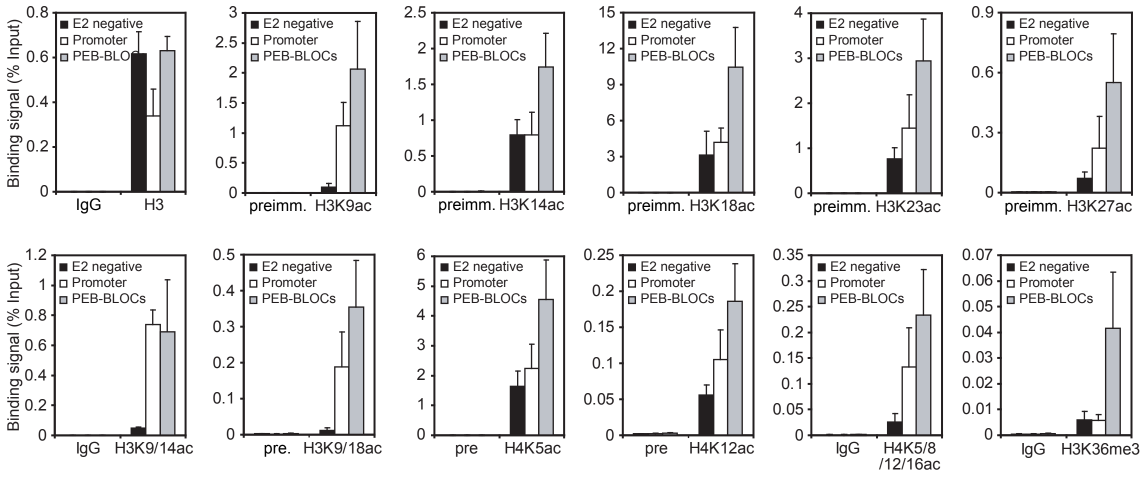

Figure S3. Persistent E2 binding regions have distinctive patterns of histone modification. Chromatin samples from mitotic HPV1 E2-expressing C-33 cells were subjected to ChIP, as shown in Figure 3F, and further analyzed with specific antibodies against histone H3, H3K9ac, H3K14ac, H3K18ac, H3K23ac, H3K27ac, H3K9ac/K14ac, H3K9ac/K18ac, H4K5ac, H4K12ac, H4K5ac/K8ac/K12ac/K16ac, and H3K36me3. ChIP DNA was analyzed by quantitative real-time PCR with primer sets for the specific E2 binding regions (see Table S3). ChIP signals were expressed as percentage of chromatin DNA immunoprecipitated from the input amount of chromatin. Average values and STDEV were calculated for 3 independent experiments on 4 non-E2 binding regions, 4 transcriptionally active promoters, and 6 persistent binding sites.
