# Supplementary material for: Papillomavirus Genomes Associate with BRD4 to Replicate at Fragile Sites in the Host Genome
Source: PLoS Pathog. 2014 May 15;10(5):e1004117. doi: 10.1371/journal.ppat.1004117 (PMC4022725; doi:10.1371/journal.ppat.1004117)
Supplement: Figure S4 — Co-localization of E2 and BRD4 and modified histones in mitotic cells. Immunofluorescence of mitotic C-33 cells expressing HPV1 E2 was performed with specific antibodies against HPV1 E2 (FLAG), BRD4, H3K56ac, H4K8ac, H3K4me1, H3K4me2, and H3K4me3. E2 protein is shown in green and BRD4 protein or the modified histones are shown in red. Cells were counterstained by DAPI (blue). (PDF) [file ppat.1004117.s004.pdf]

Figure S4

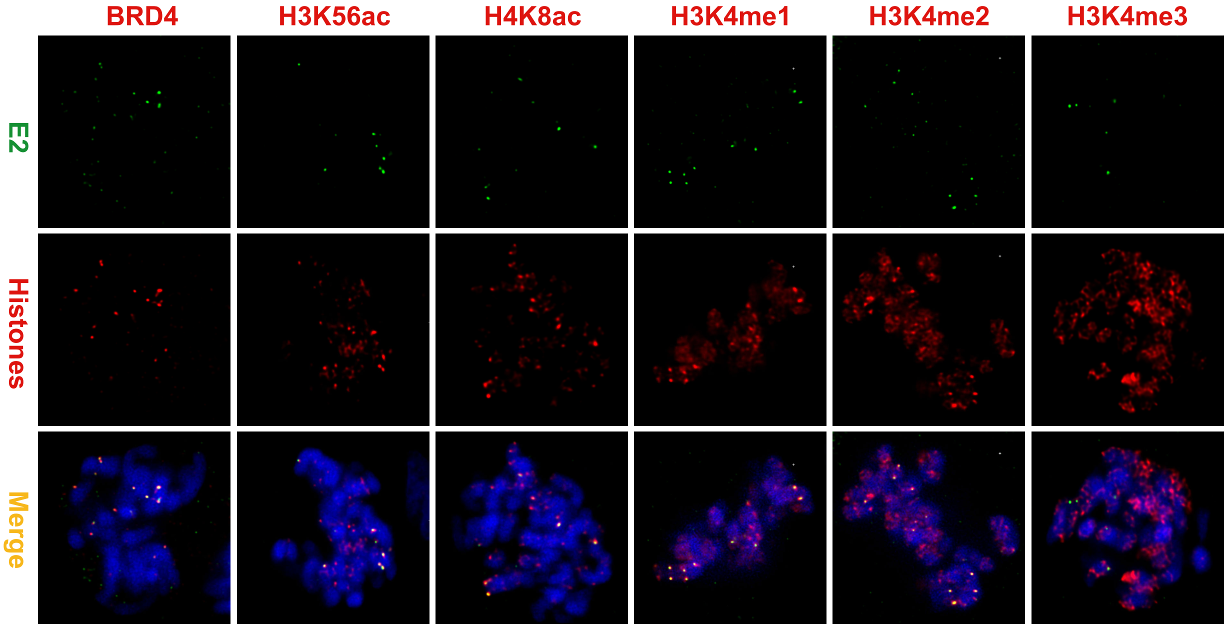

Figure S4. Co-localization of E2 and Brd4 and modified histones in mitotic cells
Immunofluorescence of mitotic C-33 cells expressing HPV1 E2 was performed with specific antibodies against HPV1 E2 (FLAG), BRD4, H3K56ac, H4K8ac, H3K4me1, H3K4me2, and H3K4me3. E2 protein is shown in green and BRD4 protein or the modified histones are shown in red. Cells were counterstained by DAPI (blue).
