# Supplementary material for: Papillomavirus Genomes Associate with BRD4 to Replicate at Fragile Sites in the Host Genome
Source: PLoS Pathog. 2014 May 15;10(5):e1004117. doi: 10.1371/journal.ppat.1004117 (PMC4022725; doi:10.1371/journal.ppat.1004117)
Supplement: Figure S5 — BRD4 colocalizes with PEB-BLOCs in interphase nuclei. C-33-1E2 cells were stained by immunofluorescence with an anti-BRD4 antibody followed by fixation. The cells were subsequently hybridized with specific FISH probes for 12 individual PEB-BLOCs. The chromosomal position of each PEB-BLOC and number of the FISH probe is shown above each panel. Image stacks were deconvolved using Huygens Essential software. Signals from IF and FISH were detected and are shown in red (BRD4) and green (FISH). The nuclei (stained with DAPI; not shown) are outlined in blue. (PDF) [file ppat.1004117.s005.pdf]

Figure S5

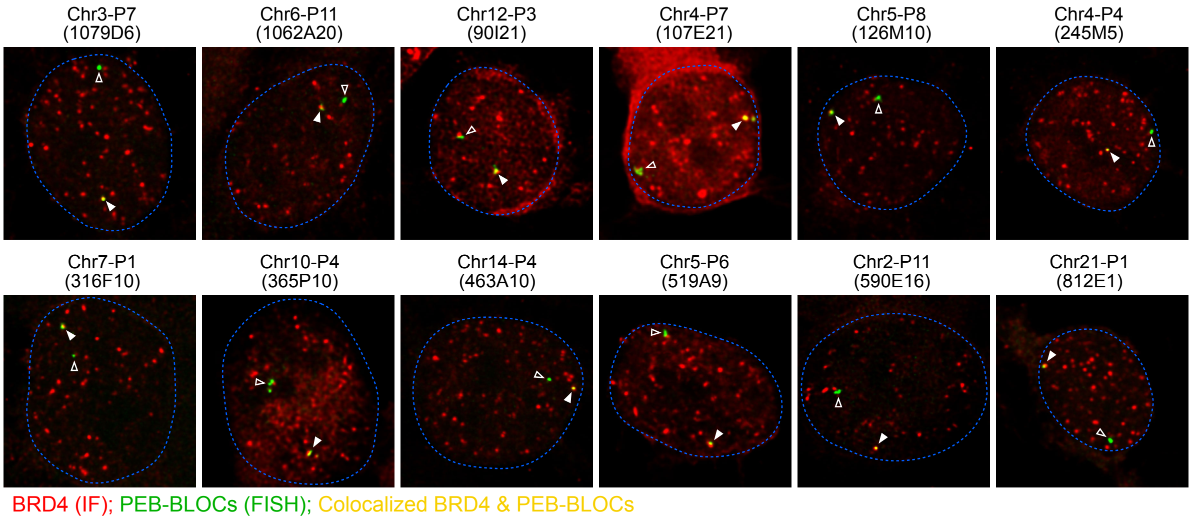

Figure S5. BRD4 colocalizes with PEB-BLOCs in interphase nuclei
C-33-1E2 cells were stained by immunofluorescence with an anti-BRD4 antibody followed by fixation. The cells were subsequently hybridized with specific FISH probes for 12 individual PEB-BLOCs. Image stacks were deconvolved using Huygens Essential software. Signals from IF and FISH were detected and are shown in red (BRD4) and green (FISH).
