# Supplementary material for: Papillomavirus Genomes Associate with BRD4 to Replicate at Fragile Sites in the Host Genome
Source: PLoS Pathog. 2014 May 15;10(5):e1004117. doi: 10.1371/journal.ppat.1004117 (PMC4022725; doi:10.1371/journal.ppat.1004117)
Supplement: Figure S6 — Deletions are common in PEB-BLOCs. A selection of eight regions (4 PEB-BLOCs in the top row and 4 FANCD2 binding sites in the bottom row) containing deletions in C-33 cells. The BRD4 in C-33 cells (BRD4con), C-33 cells expressing E2 (BRD4E2) and FANCD2 binding signals are shown. The deletions are underscored with a red line. (PDF) [file ppat.1004117.s006.pdf]

Figure S6

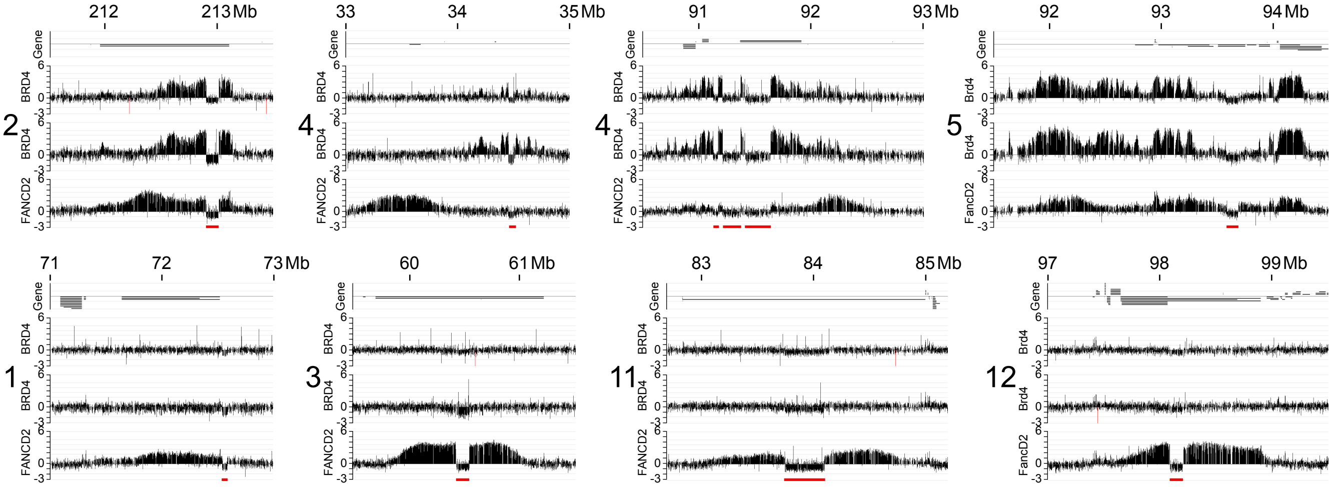

Figure S6. Deletions are common in PEB-BLOCs.

A selection of eight regions (4 PEB-BLOCs and 4 FANCD2 binding sites) containing deletions in C-33 cells. The BRD4 (in C-33 and C-33 expressing E2) and FANCD2 binding signals are shown.
