# Supplementary material for: Papillomavirus Genomes Associate with BRD4 to Replicate at Fragile Sites in the Host Genome
Source: PLoS Pathog. 2014 May 15;10(5):e1004117. doi: 10.1371/journal.ppat.1004117 (PMC4022725; doi:10.1371/journal.ppat.1004117)
Supplement: Figure S7 — FANCD2 binding in aphidicolin treated C-33 cells and E1 binding in HPV16 E1/E2 expressing cells. C-33 cells were treated with 0.2 µM aphidicolin for 24 h to cause mild replication stress before chromatin isolation. Alternatively, C-33 cells were transiently transfected with pMEP9-HPV16 E1 and pMEP4-HPV16 E2 for 24 h and treated with 3 µM CdSO4 for 4 h before harvesting. ChIP was performed using an anti-FANCD2 antibody or anti-EE antibody, respectively. ChIP-chip was performed as described for Materials and Methods. The chromosomal nucleotide positions are shown along the top. The Y-axis corresponds to a scaled log2-ratio of binding signal for FANCD2 or HPV16 E1 to input signal. The binding profiles are aligned with previously obtained BRD4 signals in the presence of E2 expression (Figure S2) for the entire set of human chromosomes. Enriched regions of BRD4 binding, FANCD2 binding and HPV16 E1 binding were defined computationally and are shown in red, and listed in Table S5. (PDF) [file ppat.1004117.s007.pdf]

Figure S7

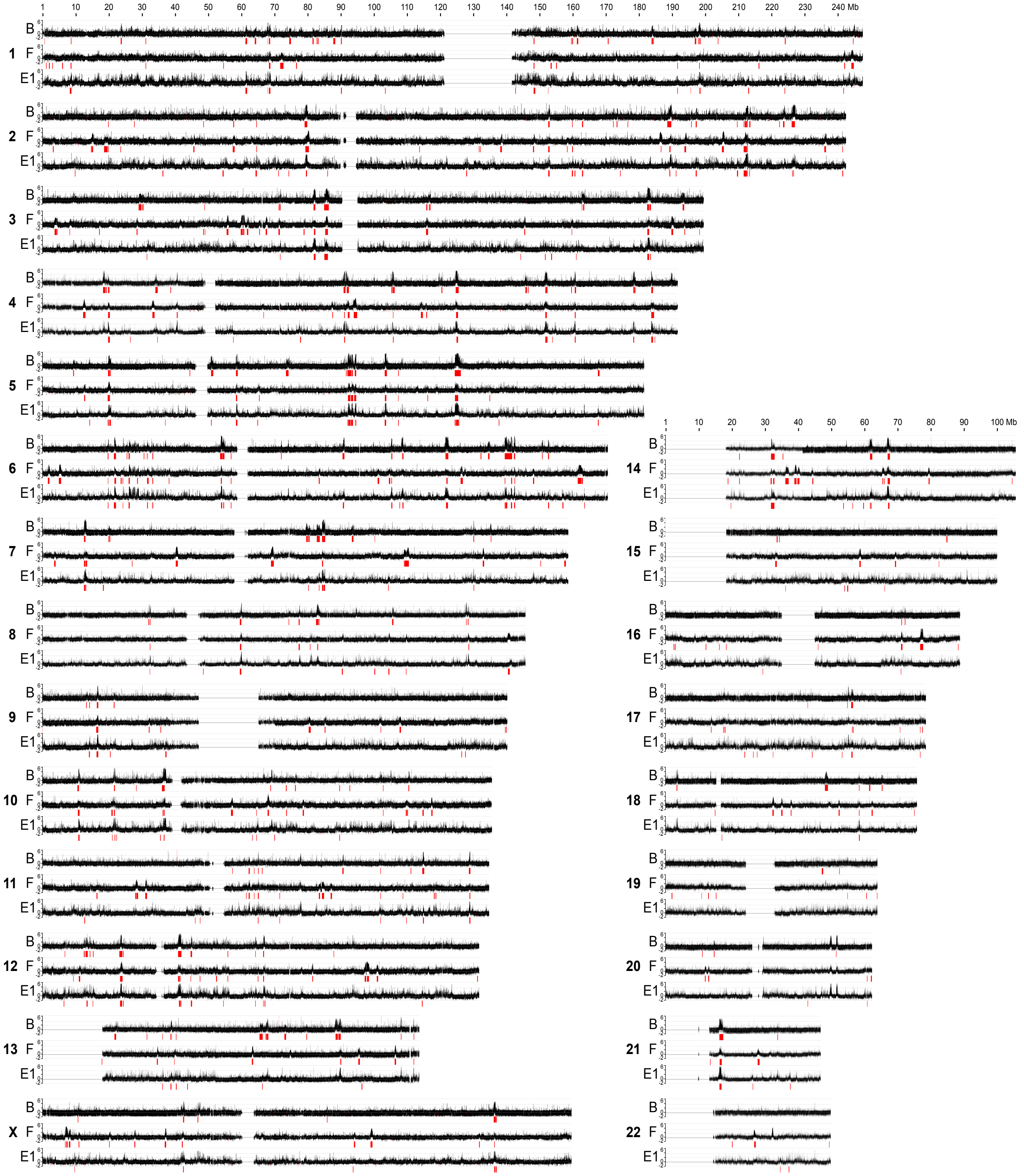


**Figure S7. FANCD2 binding in aphidicolin treated C-33 cells and E1 binding in HPV16 E1/E2 expressing cells.** C-33 cells were treated with 0.2 μM aphidicolin for 24 h to cause mild replication stress before chromatin isolation. Alternatively, C-33 cells were transiently transfected with pMEP9-HPV16 E1 and pMEP4-HPV16 E2 for 24 h and treated with 3 μM $CdSO_4$ for 4 h before harvesting. ChIP was performed using an anti-FANCD2 antibody or anti-EE antibody, respectively. ChIP-chip was performed as described for Materials and Methods. The chromosomal nucleotide positions are shown along the top. The Y-axis corresponds to a scaled log2-ratio of binding signal for FANCD2 or HPV16 E1 to input signal. The binding profiles are aligned with previously obtained BRD4 signals in the presence of E2 expression (Figure S2) for the entire set of human chromosomes. B:BRD4; F:FANCD2; E1: E1. Enriched regions of BRD4 binding, FANCD2 binding and HPV16 E1 binding were defined computationally and are shown in red, and listed in Table S5.
