# Supplementary material for: Papillomavirus Genomes Associate with BRD4 to Replicate at Fragile Sites in the Host Genome
Source: PLoS Pathog. 2014 May 15;10(5):e1004117. doi: 10.1371/journal.ppat.1004117 (PMC4022725; doi:10.1371/journal.ppat.1004117)
Supplement: Figure S8 — Histograms of nearest distance for common fragile sites and enriched regions of BRD4 and FANCD2 binding. Absolute distance tests were performed to determine whether the distance between enriched regions of BRD4 and FANCD2 binding are closer to common fragile sites than to randomly distributed regions. The common fragile sites are those mapped in epithelial cells by [50] and are listed in Table S6. A. Histogram of nearest distance for PEB-BLOCs and cFRA. B. Histogram of nearest distance for FANCD2 enriched regions and cFRA. (PDF) [file ppat.1004117.s008.pdf]

Figure S8

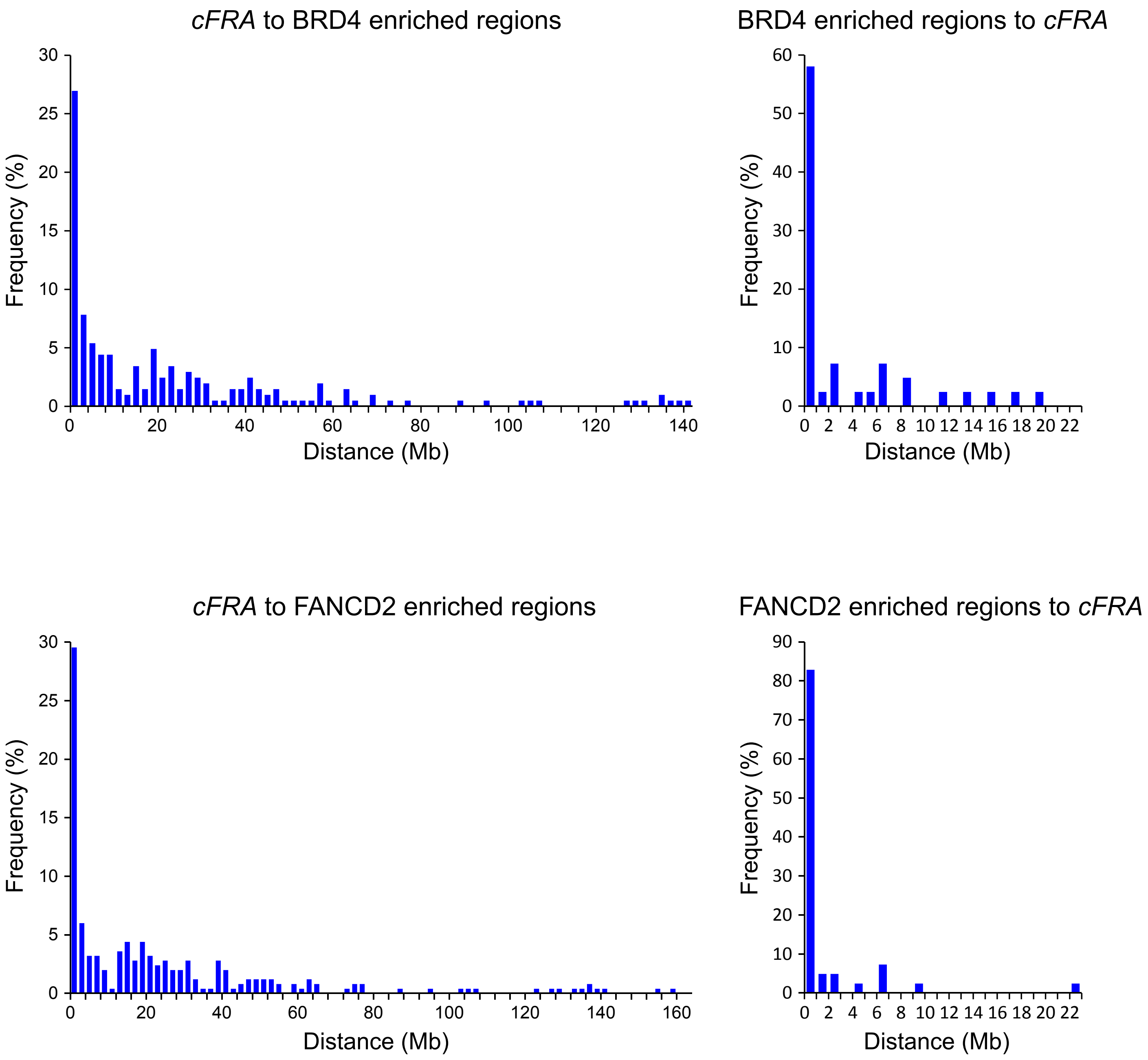

Figure S8. Histograms of nearest distance for common fragile sites and enriched regions of BRD4 and FANCD2 binding.

Absolute distance tests were performed to determine whether the distance between enriched regions of BRD4 and FANCD2 binding are closer to common fragile sites than to randomly distributed regions. The common fragile sites are those mapped in epithelial cells by (Le Tallec et al., 2013) and are listed in Table S6.

A. Histogram of nearest distance for PEB-BLOCs and cFRA

B. Histogram of nearest distance for FANCD2 enriched regions and cFRA
