# Supplementary material for: Papillomavirus Genomes Associate with BRD4 to Replicate at Fragile Sites in the Host Genome
Source: PLoS Pathog. 2014 May 15;10(5):e1004117. doi: 10.1371/journal.ppat.1004117 (PMC4022725; doi:10.1371/journal.ppat.1004117)
Supplement: Figure S9 — Papillomavirus virus genomes are often associated with PEB-BLOCs. C-33 cells were transfected with HPV1, HPV16 or HPV18 viral genomes, which were detected by FISH five days post-transfection. PEB-BLOCs were detected using BAC clones (1062A20 and 124A13 in green) corresponding to PEB-BLOCs (Chr2-P6 and Chr6-P11) and viral DNA is shown (red). A representative image is shown for each HPV with the specific PEB-BLOC region indicated. Only a small percentage of replication foci show association with individual PEB-BLOC probes, but this is consistent with the hypothesis that each focus associates with only a few PEB-BLOCs. (PDF) [file ppat.1004117.s009.pdf]

Figure S9

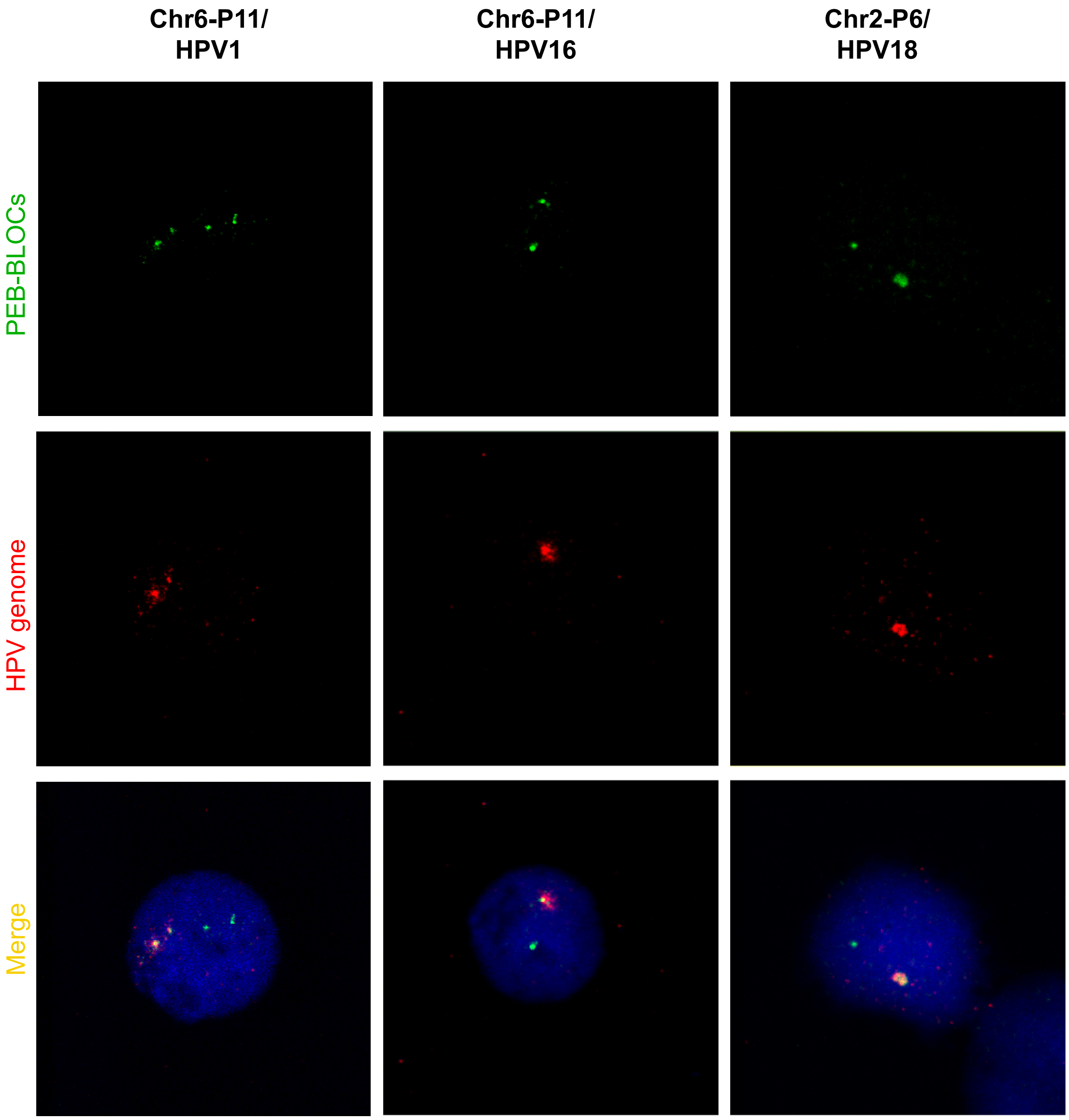

Figure S9. Papillomavirus virus genomes are often associated with PEB-BLOCs
C-33 cells were transfected with HPV1, HPV16 or HPV18 viral genomes, which were detected by FISH five days post-transfection. PEB-BLOCs were detected using BAC clones (1062A20 and 124A13 in green) corresponding to PEB-BLOCs (Chr2-P6 and Chr6-P11) and viral DNA is shown (red). A representative image is shown for each HPV with the specific PEB-BLOC region indicated. Only a small percentage of replication foci show association with individual PEB-BLOC probes, but this is consistent with the hypothesis that each focus associates with only a few PEB-BLOCs.
