# Supplementary material for: Papillomavirus Genomes Associate with BRD4 to Replicate at Fragile Sites in the Host Genome
Source: PLoS Pathog. 2014 May 15;10(5):e1004117. doi: 10.1371/journal.ppat.1004117 (PMC4022725; doi:10.1371/journal.ppat.1004117)
Supplement: Table S1 — Characteristics of PEB-BLOCs. (PDF) [file ppat.1004117.s010.pdf]

	PEB-BLOCs	Images for PEB-BLOCs	Location (hg19)	Chromosome Bands	Brd4 binding in absence of E2	Brd4 binding in presence of E2	FANCD2 binding	Genes (HuGO)	Size of Genes (Entrez gene; MBp)	Viral integration (Dr. VIS)		Fragile Sites (from HUGO)		
**Chromosome 1**														
1	**Chr1-P8**	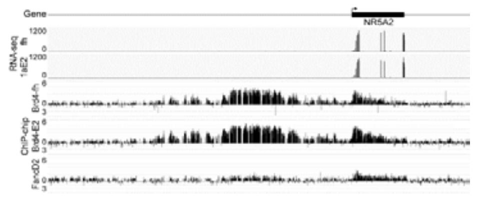	chr1:198483377-200153377	1q31.3-32.1	+++	++++	+	*NR5A2*	**194,765**	**HPV18**	**1q31**	1q31	*FRA1K*	fragile site, aphidicolin type, common, fra(1)(q31)
										**HPV18**	**1q31.1**			
										**HPV16**	**1q32**			
										**HPV16**	**1q32.1**			
										**HPV18**	**1q32.2**			
										**HPV16**	**1q32.2**			
										**HPV18**	**1q32.2-q41**			
**Chromosome 2**														
2	**Chr2-P3**	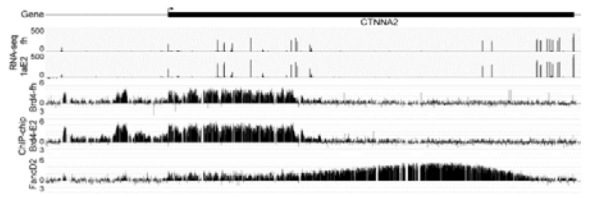	chr2:79446492-80176489	2p12	+++	++++	++++	*CTNNA2*	1,477,083	HPV16	2p13.1	2p13	*FRA2E*	fragile site, aphidicolin type, common, fra(2)(p13)
3	**Chr2-P4**	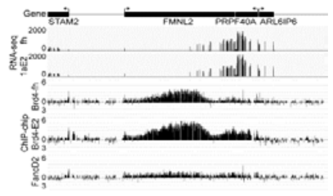	chr2:153192068-153572068	2q23.3	+++	++++	+/-	*FMNL2*	409,080	**HPV16**	**2q22**	2q22.3	*FRA2K*	fragile site, folic acid type, rare, fra(2)(q22.3)
								PRPF40A	85,650	**HPV18**	**2q22.1**			
										**HPV16**	**2q22.3**			
										HPV16	2q23			
										HPV16	2q24			
										**HPV18**	**2q24.2**			
4	**Chr2-P6**	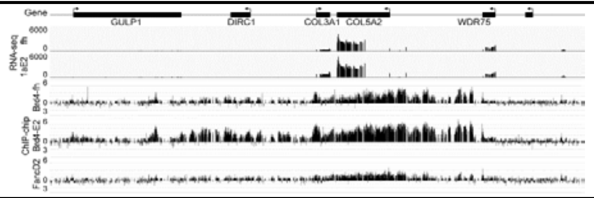	chr2:189391755 -190291755	2q32.1-32.2	++	++++	+	*GULP1*	**395,634**	HPV16	2q32	2q31	*FRA2G*	fragile site, aphidicolin type, common, fra(2)(q31)
								DIRC1	73,295	HPV18	2q32.1	2q32.1	*FRA2H*	fragile site, aphidicolin type, common, fra(2)(q32.1)
								COL3A1	49,898	HPV31	2q32.2	2q33	*FRA2I*	fragile site, aphidicolin type, common, fra(2)(q33)
								COL5A2	192,403	**HPV16**	**2q33**			
										**HPV16**	**2q33.1**			
5	**Chr2-P8**	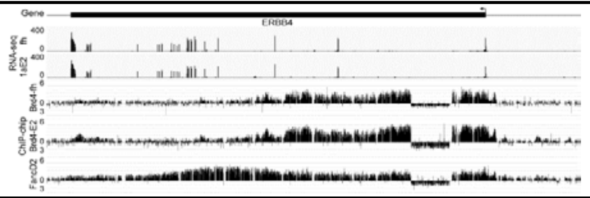	chr2:212741755-213441755	2q34	+++	++++	++++	*ERBB4*	1,512,162	HPV18	2q34	2q33	*FRA2I*	fragile site, aphidicolin type, common, fra(2)(q33)
										HPV16	2q34			
6	**Chr2-P11**	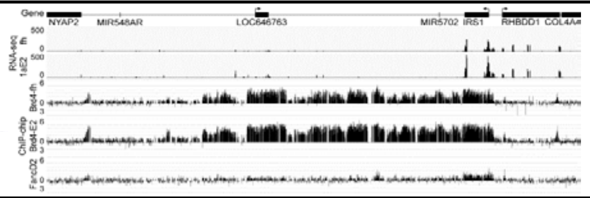	chr2:226751756 -227681756	2q36.3	+++	++++	+/-	*BC017935**	37,269			2q37.3	*FRA2J*	fragile site, aphidicolin type, common, fra(2)(q37.3)
								IRS1	87,737					
**Chromosome 3**														
7	**Chr3-P3**	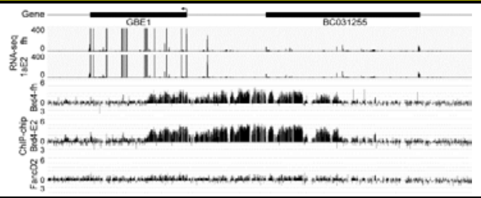	chr3:81697310-82247310	3p12.3-12.2	+++	++++	-	*GBE1*	353,819					
								*BC031255**	477,538					
8	**Chr3-P4**	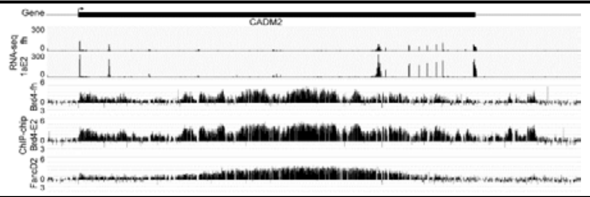	chr3:84997310-86317310	3p12.1	+++	++++	+++	*CADM2*	1,450,453					
9	**Chr3-P7**	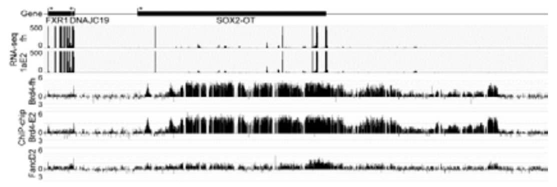	chr3:180919306-181987306	3q26.33	+++	++++	++	*SOX2-OT*	567,082	**HPV16**	**3q26**	3q25	*FRA3D*	fragile site, aphidicolin type, common, fra(3)(q25)
										HPV16	3q26.2	3q27	*FRA3C*	fragile site, aphidicolin type, common, fra(3)(q27)
										HPV16	3q26.31			
										HPV16	3q26.33	3q27	*FRA3C*	fragile site, aphidicolin type, common, fra(3)(q27)
										HPV16	3q26.33			
										**HPV16**	**3q26.33**			
										**HPV16**	**3q26.33**			
										HPV16	3q27			
										HPV16	3q27			
**Chromosome 4**														
													FRA4D	fragile site, aphidicolin type, common, fra(4)(p15)
10	**Chr4-P1**	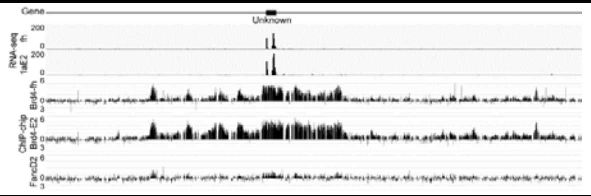	chr4:18680902-20610902	4p15.32-15.31	+++	++++	+/-	*unknown*	26,667	**HPV18**	**4p15**	4p15		
										**HPV18**	**4p15.31**	4p16.1	*FRA4A*	fragile site, aphidicolin type, common, fra(4)(p16.1)
										HPV16	4p16			
11	**Chr4-P4**	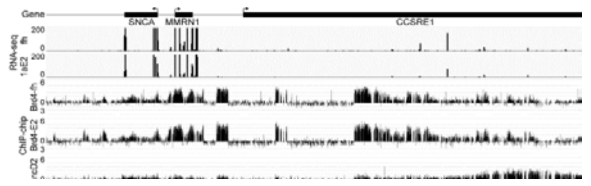	chr4:90640977-92040977	4q22.1	+++	++++	++	*SNCA*	148,495	HPV18	4q21	4q22	FRA4F	fragile site, aphidicolin type, common, fra(4)(q22)
								MMRN1	77,666	**HPV16**	**4q21**			
								CCSRE1	1,917,572	**HPV16**	**4q21.21**			
										**HPV16**	**4q21.23**			
										HPV16	4q23			

										HPV16	4q21.22			
12	**Chr4-P5**	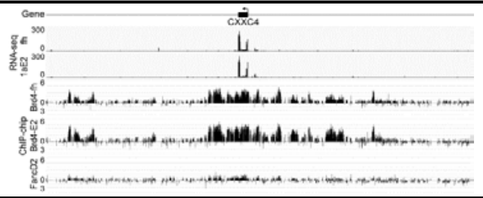	chr4:105280551-105810551	4q24	+++	++++	+/-	_CXXC4_<br>_AK094561*_	34,582<br>185,224	HPV16	4q23			
13	**Chr4-P7**	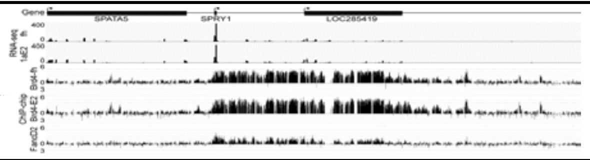	chr4:124310550-125040550	4q28.1	++++	++++	+	_SPRY1_<br>_LOC285149*_	9,058<br>277,577	HPV18	4q28.3	4q28.1	_KIAA1109_	FLJ21404, "fragile site-associated", FSA
14	**Chr4-P9**	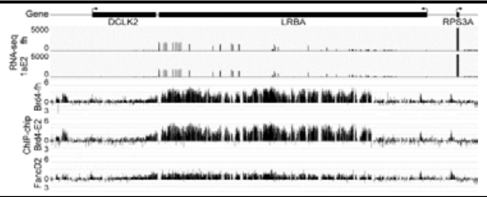	chr4:151160550-151780550	4q31.3	++++	++++	+	_LRBA_	976,334	**HPV16**<br>**HPV16**	**4q31**<br>**4q31.21**	4q31.1	_FRA4C_	fragile site, aphidicolin type, common, fra(4)(q31.1)
15	**Chr4-P10**	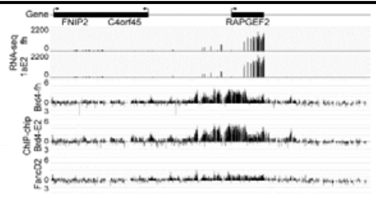	chr4:158910550-160280550	4q32.1	+++	+++	**+/-**	_RAPGEF2_	120,026			4q31.1	_FRA4C_	fragile site, aphidicolin type, common, fra(4)(q31.1)
16	**Chr4-P11**	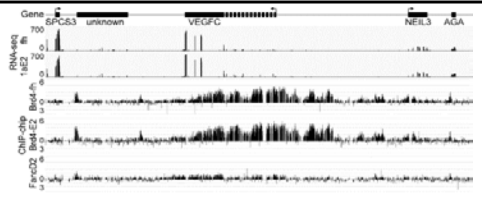	chr4:177623006-178033006	4q34.3	+++	++++	+/-	_VEGFC_<br>_unknown_	142,002<br>142,883					
17	**Chr4-P12**	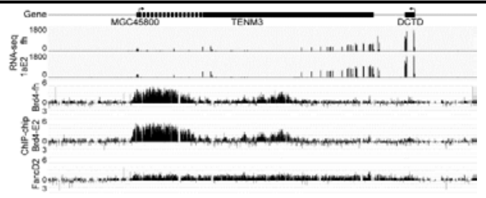	chr4:183053006-183513006	4q35.1	+++	++++	++	_TENM3_	622,908	HPV16	4q35.2			
**Chromosome 5**														
18	**Chr5-P2**	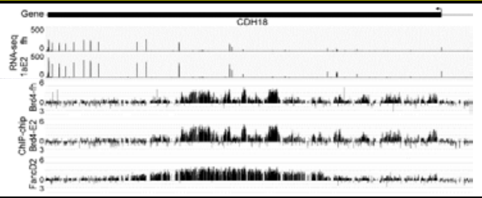	chr5:19854243-20579243	5p14.3	+++	+++	+++	_CDH18_	1,108,275	HPV33<br>HPV16<br>**HPV18**<br>HPV16	5p14<br>5p14.1<br>**5p15**<br>5p15.3	5p13<br>5p14	_FRA5A_<br>_FRA5E_	fragile site, BrdU type, common, fra(5)(p13)<br>fragile site, aphidicolin type, common, fra(5)(p14)
19	**Chr5-P3**	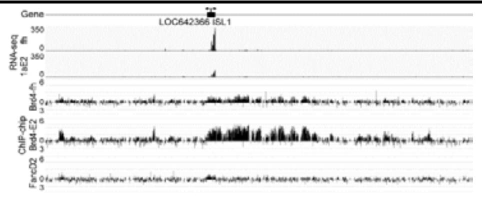	chr5:50674243-50984243	5q11.1-11.2	++	++++	-	_LOC642366_<br>_ISL1_	10,596<br>15,092	 HPV16	5q11.1<br>5q11.2			
20	**Chr5-P4**	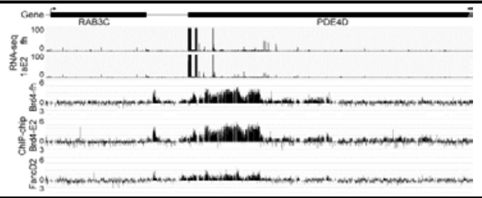	chr5:58264243-58494243	5q11.2	+++	++++	++	_PDE4D_	1,975,272	HPV16	5q11.2			
21	**Chr5-P6**	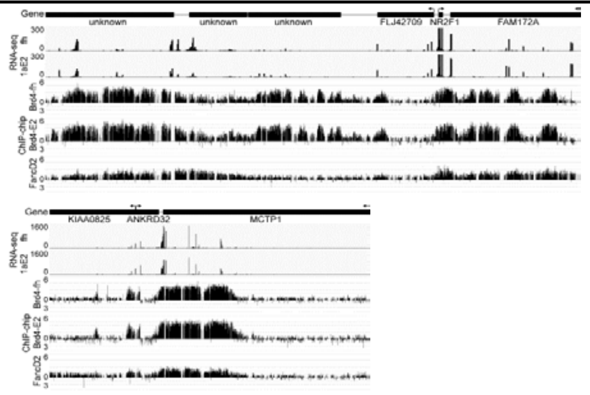	chr5:91694244-94284244	5q14.3-15	+++	++++	+++	_unknown_<br>_FLJ42709*_<br>_NR2F1_<br>_FAM172A_<br>_KIAA0825_<br>_ANKRD32_<br>_MCTP1_	816,741<br>154,105<br>13,973<br>642,236<br>467,754<br>100,362<br>751,581	**HPV18**	**5q15**	5q15<br>5q15	_FRA5B_<br>_FRA5D_	fragile site, BrdU type, common, fra(5)(q15)<br>fragile site, aphidicolin type, common, fra(5)(q15)
22	**Chr5-P7**	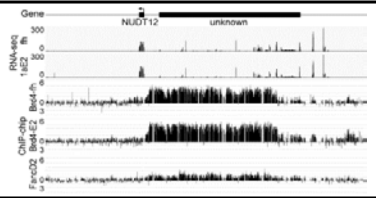	chr5:102902101-103302101	5q21.2	++++	++++	+	_unknown_	431,591			 5q21	_FRA5F_	fragile site, aphidicolin type, common, fra(5)(q21)
23	**Chr5-P8**	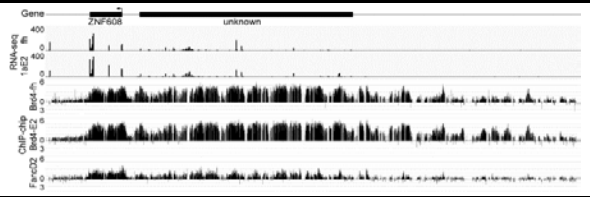	chr5:123942101-125482101	5q23.2	+++	++++	++	_ZNF608_<br>_unknown_	307,775<br>709,784					
**Chromosome 6**														
24	**Chr6-P2**	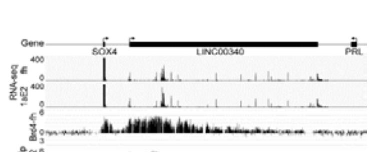	chr6:21582021-22152021	6p22.3	+++	+++	++	_SOX4_<br>_LINC00340_	6,344<br>686,512	**HPV16**<br>**HPV16**<br>**HPV16**<br>HPV18	**6p21.2**<br>**6p21.2**<br>**6p21.31**<br>6p22.1	6p22.2<br>6p23	_FRA6C_<br>_FRA6A_	fragile site, aphidicolin type, common, fra(6)(p22.2)<br>fragile site, folic acid type, rare, fra(6)(p23)

		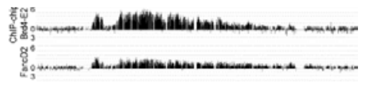								**HPV16**	**6p22.2**			
										**HPV16**	**6p22.3**			
										HPV18	6p23			
25	**Chr6-P6**	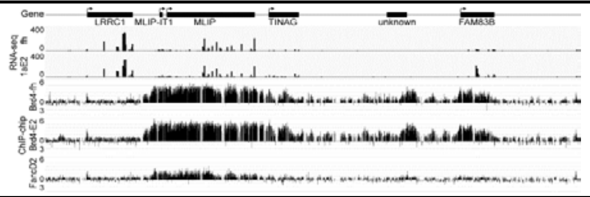	chr6:53812041-54832041	6p12.1	++++	++++	++	*MLIP*	321,655	**HPV16**	**6p12**			
								TINAG	106,295	**HPV16**	**6p12.2**			
								unknown	99,296					
								FAM83B	131,468					
26	**Chr6-P8**	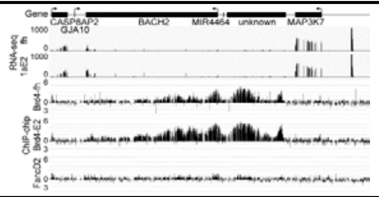	chr6:90753279-91193279	6q15	++	++++	+/-	*BACH2*	481,615	HPV33	6q15	6q15	*FRA6G*	fragile site, aphidicolin type, common, fra(6)(q15)
								unknown	192,646					
27	**Chr6-P9**	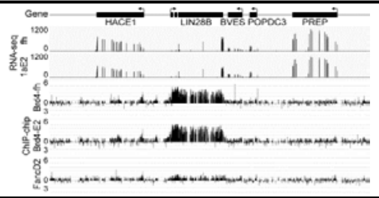	chr6:105373307-105543307	6q21	+++	+++	+/-	*LIN28B*	164,212	**HPV16**	**6q21**	6q21	*FRA6F*	fragile site, aphidicolin type, common, fra(6)(q21)
28	**Chr6-P10**	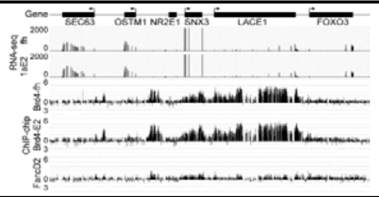	chr6:108423307-108893307	6q21	++++	++++	+/-	*NR2E1*	29,646	**HPV16**	**6q21**	6q21	*FRA6F*	fragile site, aphidicolin type, common, fra(6)(q21)
								SNX3	64,688					
								LACE1	296,683					
29	**Chr6-P11**	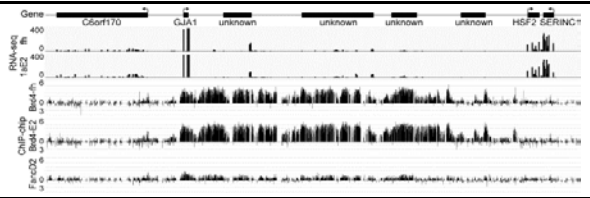	chr6:121728301-122698301	6q22.31	++++	++++	+	*GJA1*	18,371	HPV16	6q22.31	6q21	*FRA6F*	fragile site, aphidicolin type, common, fra(6)(q21)
								unknown	76,545	HPV16	6q22.31			
								unknown	206,804					
								unknown	77,888					
30	**Chr6-P13**	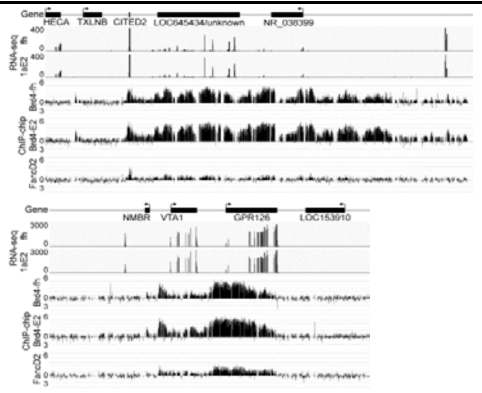	chr6:139658307-142808307	6q24.1 -24.2	+++	++++	+	*CITED2*	3,114	HPV16	6q23.3			
								LOC645434	5,602	HPV16	6q23.3			
								unknown	140,728	HPV16	6q25			
								LOC100132735	89,399					
								*BC038188**	117,392					
								NMBR	17,154					
								VTA1	95,801					
								GPR126	187,698					
**Chromosome 7**														
31	**Chr7-P1**	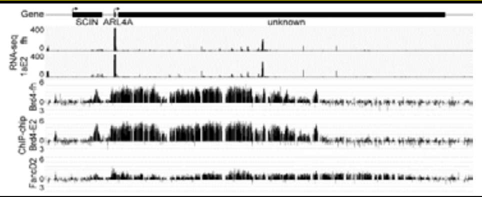	chr7:12653475-13333475	7p21.3	++++	++++	++	*SCIN*	107,960	HPV16	7p22	7p22	*FRA7B*	fragile site, aphidicolin type, common, fra(7)(p22)
								ARL4A	5,341	HPV18	7p22.1			
								unknown	916,170					
										HPV16	7q21			
32	**Chr7-P4**	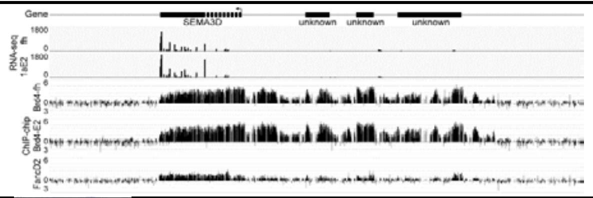	chr7:84612064-85572064	7q21.11	++++	++++	+	*SEMA3D*	126,378	HPV16	7q21	7q21.2	*FRA7E*	fragile site, aphidicolin type, common, fra(7)(q21.2)
								unknown	200,098			7q22	*FRA7F*	fragile site, aphidicolin type, common, fra(7)(q22)
**Chromosome 8**														
33	**Chr8-P3**	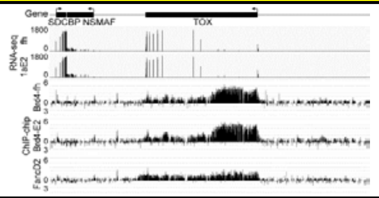	chr8:59707446-60037446	8q12.1	+++	++++	++	*TOX*	408,030	**HPV16**	**8q12.1**			
										HPV16	8q13			
34	**Chr8-P5**	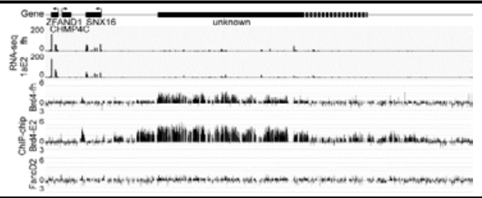	chr8:82787445-83677445	8q21.13	++	++++	-	*unknown*	411,493	**HPV18**	**8q21**	8q22.1	*FRA8B*	fragile site, aphidicolin type, common, fra(8)(q22.1)
										**HPV18**	**8q21.2**	8q22.3	*FRA8A*	fragile site, folic acid type, rare, fra(8)(q22.3)
										HPV16	8q21.3			
										HPV33	8q22.3			
35	**Chr8-P8**	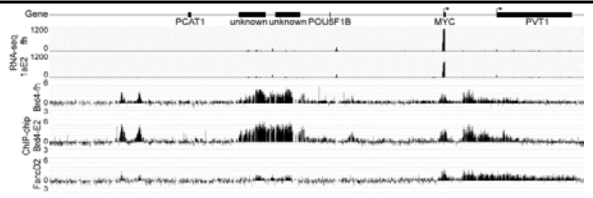	chr8:127830818-128930818	8q24.21	++	++++	++	*unknown*	97,815	HPV18	8q23.3-q24	8q24.1	*FRA8C*	fragile site, aphidicolin type, common, fra(8)(q24.1)
								unknown	90,479	**HPV18**	**8q24**	8q24.1	*FRA8E*	fragile site, distamycin A type, rare, fra(8)(q24.1)
								POU5F1B	2,079	**HPV16**	**8q24**	8q24.3	*FRA8D*	fragile site, aphidicolin type, common, fra(8)(q24.3)
								MYC	6,977	**HPV18**	**8q24.1**			
								PVT1	273,882	**HPV16**	**8q24.1**			
										**HPV16**	**8q24.1**			
										**HPV18**	**8q24.12**			
										**HPV18**	**8q24.2**			
										**HPV18**	**8q24.21**			
										**HPV16**	**8q24.21**			
										**HPV16**	**8q24.21**			

										**HPV16**<br>HPV16<br>**HPV18**<br>**HPV16**	**8q24.21**<br>8q24.21<br>**8q24.3**<br>**8q24.3**			
**Chromosome 9**														
36	**Chr9-P1**	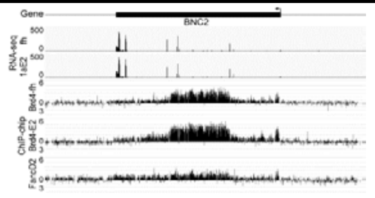	chr9:16410000-16870000	9p22.3/22.2	+++	++++	+	*BNC2*	599,821	HPV16<br>HPV16<br>HPV16<br>HPV16	9p22<br>9p23<br>9q22.32<br>9q22.32	9p21<br>9p21	*FRA9A*<br>*FRA9C*	fragile site, folic acid type, rare, fra(9)(p21)<br>fragile site, BrdU type, common, fra(9)(p21)
**Chromosome 10**														
37	**Chr10-P1**	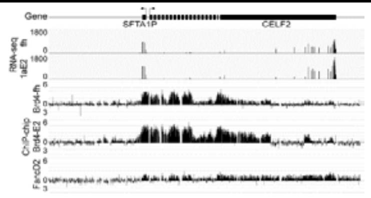	chr10:10809994-11199994	10p14	+++	++++	+	*LOC254312**<br>*CELF2*	17,223<br>430,947	HPV16	10p15			
38	**Chr10-P2**	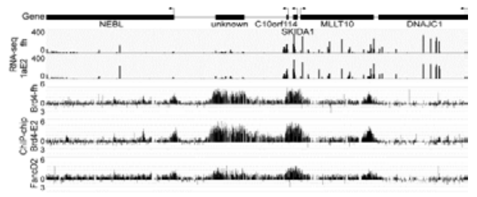	chr10:21539994-21839994	10p12.31	+++	+++	++	*unknown*<br>*C10orf114*<br>*SKIDA1*	83,796<br>3,631<br>15,870					
39	**Chr10-P4**	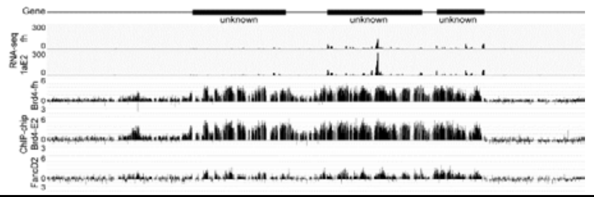	chr10:35899994-37189994	10p11.21	+++	++++	+	*unknown*<br>*unknown*<br>*unknown*	276,439<br>263,582<br>133,871	**HTLV1**<br>**HTLV1**				
**Chromosome 11**														
40	**Chr11-P4**	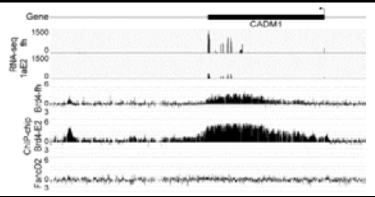	chr11:114994790-115374790	11q23.2	++	++++	-	*CADM1*	430,272	HPV45<br>HPV16<br>HPV16<br>HPV18	11q22.2<br>11q23<br>11q23-q25<br>11q23.3	11q23.3<br>11q23.3	*FRA11B*<br>*FRA11G*	fragile site, folic acid type, rare, fra(11)(q23.3)<br>fragile site, aphidicolin type, common, fra(11)(q23.3)
41	**Chr11-P5**	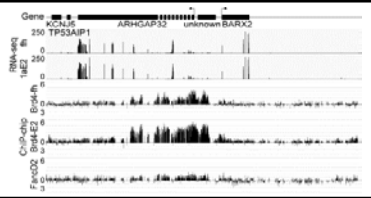	chr11:128984790-129214790	11q24.3	++	++++	+	*ARHGAP32*<br>*AK127847**	295,534<br>49,386	HPV16<br>HPV16	11q23<br>11q23-q25	11q23.3<br>11q23.3	*FRA11B*<br>*FRA11G*	fragile site, folic acid type, rare, fra(11)(q23.3)<br>fragile site, aphidicolin type, common, fra(11)(q23.3)
**Chromosome 12**														
42	**Chr12-P2**	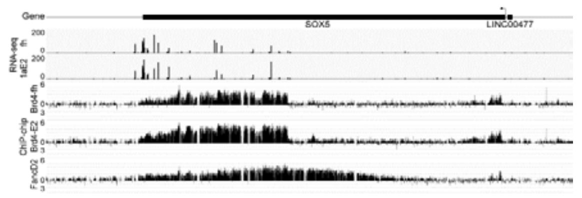	chr12:23668733-24108733	12p12.1	+++	++++	+++	*SOX5*	1,339,533	HPV18<br>HPV16<br>HPV16	12p11.2<br>12p11.2<br>12p12			
43	**Chr12-P3**	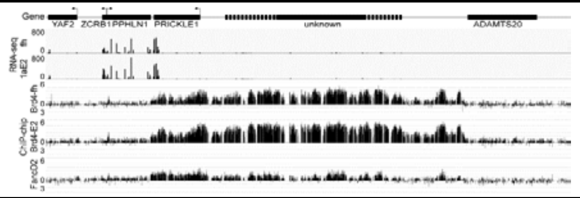	chr12:42823733-43763733	12q12	+++	++++	++	*PRICKLE1*<br>*unknown*	170,905<br>321,900	HPV16<br>HPV18<br>HPV16<br>HPV18	12q12<br>12q13<br>12q13<br>12q13-q15	12q13.1	*FRA12A*	fragile site, folic acid type, rare, fra(12)(q13.1)
44	**Chr12-P7**	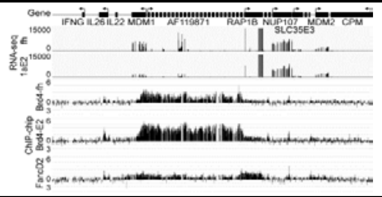	chr12:68713733-69013733	12q15	++	++++	+	*MDM1*<br>*AF119871 **<br>*RAP1B*	49,172<br>454,104<br>64,713	HPV18<br>HPV18<br>**HPV16**	12q13-q15<br>12q14.3<br>**12q14.3**			
**Chromosome 13**														
45	**Chr13-P6**	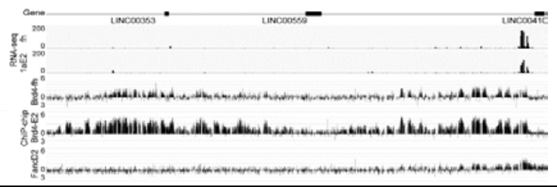	chr13:89821999-91531999	13q31.2-31.3	+	++++	+/-	*LINC00353*<br>*LINC00559*<br>*BC038529*	15,620<br>59,471<br>42,054			13q32	*FRA13D*	fragile site, aphidicolin type, common, fra(13)(q32)
**Chromosome 14**														
46	**Chr14-P1**	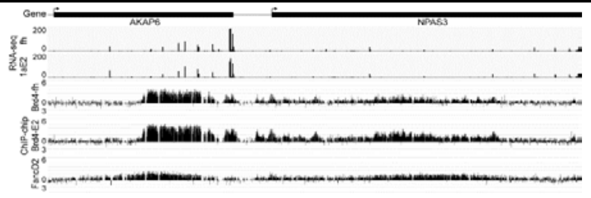	chr14:33040249-34052249	14q13.1	+++	++++	++	*AKAP6*<br>*NPAS3*	655,089<br>1,124,681	HPV18<br>HPV16	14q13<br>14q13.3			
47	**Chr14-P3**	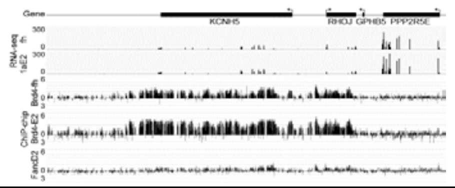	chr14:63070247-63770247	14q23.2	++	++++	+/-	*KCNH5*<br>*RHOJ*	513,160<br>115,895			14q23<br>14q24.1	*FRA14B*<br>*FRA14C*	fragile site, aphidicolin type, common, fra(14)(q23)<br>fragile site, aphidicolin type, common, fra(14)(q24.1)

48	**Chr14-P4**	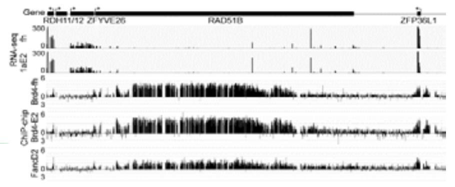	chr14:68280247-69311247	14q24.1	**++++**	**++++**	**++**	*ZFYVE26*<br>*RAD51B*<br>*ZFP36L1*	91,112<br>1,009,367<br>11,167	**HPV18**<br>**HPV45**<br>**HPV18**<br>**HPV16**<br>**HPV18**<br>**HPV16**	**14q24**<br>**14q24.1**<br>**14q24.1**<br>**14q24.1**<br>**14q24.3**<br>**14q24.3**	14q23<br>14q24.1	*FRA14B*<br>*FRA14C*	fragile site, aphidicolin type, common, fra(14)(q23)<br>fragile site, aphidicolin type, common, fra(14)(q24.1)
**Chromosome 18**														
49	**Chr18-P3**	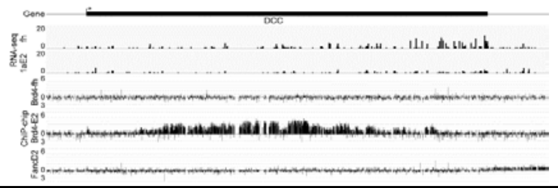	chr18:50096002-50746002	18q21.2	**-**	**+++**	**-**	*DCC*	1,554,840	HPV68<br>**HPV16**<br>HPV18<br>**HPV16**<br>**HPV16**	18q21<br>**18q21.2**<br>18q21.3<br>**18q21.31**<br>**18q21.33**	18q21.3<br>18q22.2	*FRA18B*<br>*FRA18C*	fragile site, aphidicolin type, common, fra(18)(q21.3)<br>fragile site, aphidicolin type, common, fra(18)(q22.2)
**Chromosome 20**														
50	**Chr20-P3**	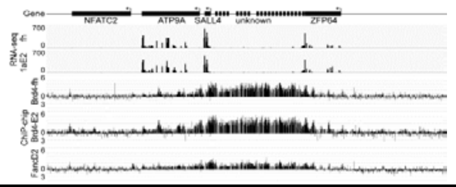	chr20:50206593-50816593	20q13.2	**+++**	**++++**	**++**	*ATP9A*<br>*SALL4*<br>*unknown*<br>*ZFP64*	223,128<br>24,013<br>52,228<br>140,401	HPV16<br>HPV45	20q13.13<br>20q13.2			
51	**Chr20-P4**	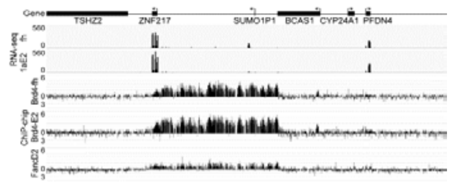	chr20:52166593-52586593	20q13.2	**+++**	**++++**	**+**	*ZNF217*<br>*SUMO1P1*	20,840<br>1,576	HPV16<br>HPV45<br>HPV16	20q13.13<br>20q13.2<br>20q13.13			
**Chromosome 21**														
52	**Chr21-P1**	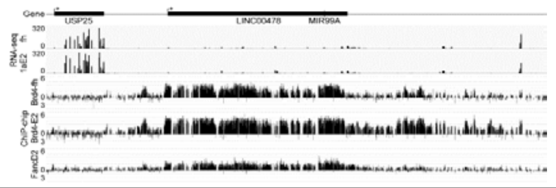	chr21:17328129-18648129	21q21.1	**+++**	**++++**	**++**	*LINC00478*	701,204	HPV18<br>HPV16<br>HPV16	21q21<br>21q21.3<br>21q22			
**Chromosome X**														
53	**ChrX-P4**	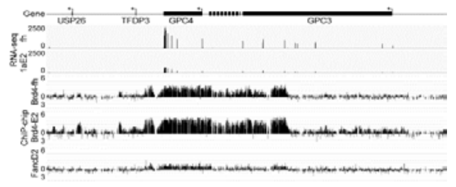	chrX:132302334-132822334	Xq26.2	**+++**	**++++**	**+/-**	*TFDP3*<br>*GPC4*<br>*GPC3*	2,184<br>148,421<br>585,017	HPV18	Xq25	Xq27.2<br>Xq27.3	*FRAXD*<br>*FRAXA*	fragile site, aphidicolin type, common, fra(X)(q27.2) D<br>fragile site, folic acid type, rare, fra(X)(q27.3) A (macroorchidism, mental retardation)
