# Supplementary material for: Papillomavirus Genomes Associate with BRD4 to Replicate at Fragile Sites in the Host Genome
Source: PLoS Pathog. 2014 May 15;10(5):e1004117. doi: 10.1371/journal.ppat.1004117 (PMC4022725; doi:10.1371/journal.ppat.1004117)
Supplement: Table S8 — List of antibodies. (PDF) [file ppat.1004117.s017.pdf]

Supplementary Table 8. Antibodies used in this study

Name	Origins	Concent-ration (µg/µl)	Used amount		
			For ChIP (µl)	For IF (Dilution)	For WB (Dilution)
Mouse IgG	Jackson ImmunoRes	5.6	0.5		
Rabbit IgG	Jackson ImmunoRes	11	0.3		
Rabbit preimmune serum	Homemade	Serum	3		
Anti-BRD4, C-term, Affinity-purified	Homemade	1	3	1:100	
Anti-BRD4, C-term (1386-1400 aa)	Homemade	Serum	3	1:250	1:2000
Anti-BRD4, N-term (169-346 aa)	Homemade	Serum	3	1:250	1:2000
Anti-FLAG M2	Sigma, F1804	Serum	3	1:500	1:5000
Anti-acetyl Histone H3 (Lys9)	Upstate, 07-352	Serum	3		
Anti-acetyl-Histone H3 (Lys14)	Upstate, 07-353	Serum	3		
Anti-acetyl-Histone H3 (Lys18)	Upstate, 07-354	Serum	3		
Anti-acetyl-Histone H3 (Lys23)	Upstate, 07-355	Serum	3		
Anti-acetyl-Histone H3 (Lys27)	Upstate, 07-360	Serum	3		
Anti-acetyl-Histone H3 (Lys56)	Upstate, 07-677<br>Epitomics 2134-1	Serum<br>R mAb	3<br>3	1:500<br>1:500	1:5000<br>1:5000
Anti-acetyl-Histone H3 (Lys9/14)	Upstate, 06-599	1	3		
Anti-acetyl-Histone H3 (Lys9/18)	Upstate, 07-593	Serum	3		
Anti-acetyl-Histone H4 (Lys5)	Upstate, 07-327	Serum	3		
Anti-acetyl-Histone H4 (Lys8)	Upstate, 07-328	Serum	3	1:500	1:5000
Anti-acetyl-Histone H4 (Lys12)	Upstate, 07-595	Serum	3		
Anti-acetyl-Histone H4 (Lys16)	Upstate, 06-762	1	3		
Anti-acetyl-Histone H4 (Lys5/8/12/16)	Upstate, 06-598	1	3		
Anti-Histone H3 (monomethyl K4)	Abcam, ab8895	0.8	3.75	1:500	1:5000
Anti-Histone H3 (dimethyl K4)	Upstate, 07-030	Serum	3	1:500	1:5000
Anti-Histone H3 (trimethyl K4)	Abcam, ab8580	0.4	7.5	1:500	1:5000
Anti-Histone H3 (monomethyl K9)	Abcam, ab9045	0.5	6		
Anti-Histone H3 (dimethyl K9)	Upstate, 07-441	1	3		
Anti-Histone H3 (trimethyl K9)	Abcam, ab8898	0.5	6		
Anti-Histone H3 (monomethyl K27)	Upstate, 07-448	1	3		
Anti-Histone H3 (trimethyl K27)	Upstate, 07-449	1	3		
Anti-Histone H3 (monomethyl K36)	Abcam, ab9048	0.5	6		
Anti-Histone H3 (trimethyl K36)	Abcam, ab9045	1	3		
Anti-Histone H3, CT, pan	Upstate, 07-690	Serum	3		1:5000
Anti- Histone H3	Abcam, ab1791	1	3.75		
Anti- CREBBP	Abcam, ab3652	1		1:200	
Anti-EP300	Abcam, ab14984	1		1:100	
Anti-KAT5	Abcam, ab62644	1		1:100	
Anti-FANCD2	Bethyl Lab, A302-174A	0.2	5	1:200	1:2000
