# Supplementary material for: Papillomavirus Genomes Associate with BRD4 to Replicate at Fragile Sites in the Host Genome
Source: PLoS Pathog. 2014 May 15;10(5):e1004117. doi: 10.1371/journal.ppat.1004117 (PMC4022725; doi:10.1371/journal.ppat.1004117)
Supplement: Table S9 — List of Q-PCR primers. (PDF) [file ppat.1004117.s018.pdf]

Supplementary Table 9. Q-PCR primers used in this study

Groups	Targets (Chr location)	Sequences (5' to 3')
PEB-BLOCs	Chr3-P3 (Chr3-81792810)	GATTCATGGGACAACGAAGTTAGA
		CCTTTCTGGATGTTCCAATTTCA
	Chr3-P4 (Chr3-85635810)	GCACGGCCTGTCTTGCA
		TCAGCAGGCTCCATTGTGAA
	Chr3-P7 (Chr3-181077800)	GACAACAATTTCAGTGCCTTTTACA
		TGTTTTCTTTCTGGACCACATAATCT
	Chr4-P1 (Chr4-19021400)	GCTGCCGTTCCGCAACT
		GCAAAAGGGAGAGCCAGAAGT
	Chr4-P4 (Chr4-90968480)	CATTCCAAGGGCCAAGGTT
		GCCCCACCTGCCTCAAC
	Chr4-P7 (Chr4-124537550)	CCTTACCATTTGCTTTCACAGATTT
		CAAGATTAGGTACTCAGCACAATGC
	Chr4-P9 (Chr4-151510050)	AACAGGCCTCAGGCTATCTAACA
		TGGCCTTGGAAGCCTATTGA
	Chr4-P12 (Chr4-183113000)	AACACCGTTGCACCCATTTT
		GTTGTCGTGTCTCTGGCATGA
	Chr5-P8 (Chr5-124746100)	TGGTGTATAAGAGCTCCCAATGAG
		CCTGGCCCTCCTTTGTCA
	Chr20-P3 (Chr20-50579090)	CGTATCTGATCATTTCTGCTTGTGT
		CCCGCCTTCCGACAAAG
	Chr20-P4 (Chr20-52354090)	ACAGCCGCCCAGATAAAGTG
		TTTCATGGCGTGAAATAACCAA
	Chr21-P1 (Chr21-17559630)	CTTGTTGAACTGGGTGCTCTTG
		GGTATCAGAATGTGCAGGTGAAAT
	Chr5-P8 (Chr5-124082100)	CAGCCAGAATAATCACTCGATAACATTA
		AGGACTGATTCGTTTGCCAAA
	Chr5-P8 (Chr5-124240100)	TGATCCGCCTGCCTTAGC
		TTCTACACACTGTGCCTACCTAAATAAAT
	Chr5-P8 (Chr5-124455100)	TTGGTGTCAGATCACAATAGGAAAC
		GGCATTTAGGAGGGATCTTTGTT
	Chr5-P8 (Chr5-124642100)	AACCAAGCGTGGGCCTTT
		AGGCATGTGACCGATCCAA
Active promoters	*BRD2*	GCAGCCATGCTGAACTCGTA
		CGCTGCTGCTTGTCCATATC
	CITED2	TCTCGCTCCGCCTTTCC
		GCTGCCAACAATGAGCTGTGT
	ID2	TCCGATGGGTTGCAGTGAA
		ACAGCTACACGGGCGAGTTC
	LIN28	TGTTTGAGTTTTTCGTGTTCATTCA
		CTGGCAAGAGGAAGAGATAACCA
	SALL4	TCCCAACTCCAGGAATTTGTG
		TCGACATGGTGCGAGCAT
	TUBB	GCGACCTGCGGAGAAAAAA
		CACGATTTCCCTCATGGTTAAAA
Negative Controls	No BRD4/E2 (Chr3-179957300)	CTTCTCCACACTCCTCACAGTCA
		AGTGGTGCTTGATAAGACACAACAG
	No BRD4/E2 (Chr4-127730550)	CCGTTAGAAAATCTTGGACCAAA
		CAAAGCCCCAGCCTTGTTAA
	No BRD4/E2 (Chr5-124117100)	GAGGAAGGTCAGAGCCATGATC
		ACCTCGAGCGTTGTCTCTTAGC
	Inactive promoter (*HOXB1*)	GTTGTAGGGCAAGAGGGTGTCT
		CATCCTATTATAGTCCATGCGTCAA
