# Supplementary material for: Papillomavirus Genomes Associate with BRD4 to Replicate at Fragile Sites in the Host Genome
Source: PLoS Pathog. 2014 May 15;10(5):e1004117. doi: 10.1371/journal.ppat.1004117 (PMC4022725; doi:10.1371/journal.ppat.1004117)
Supplement: Table S10 — List of Bac clones. (PDF) [file ppat.1004117.s019.pdf]

Supplementary Table 10. BAC clones for FISH used in this study

Targets		BAC clones (RPCI-11)	Chromosome location
PEB-BLOCs	Chr2-P6	124A13	chr2: 189,651,111 - 189,804,889
	Chr2-P11	590E16	chr2: 227,045,563 – 227,234,560
	Chr3-P4	452E16	chr3: 85,538,607 - 85,709,161
	Chr3-P7	275H4	chr3: 180,985,007 - 181,139,502
		1079D6	chr3: 181,191,777 - 181,383,072
	Chr4-P4	245M5	chr4: 90,971,482 - 91,132,082
		451M10	chr4: 91,618,396 - 91,768,647
	Chr4-P7	107E21	chr4: 124,676,237 - 124,846,274
	Chr5-P6	519A9	chr5: 92,951,412 – 93,152,841
	Chr5-P8	126M10	chr5: 124,455,878 - 124,620,982
		209F21	chr5: 124,758,641 - 124,941,975
	Chr6-P11	1062A20	chr6: 122,019,915 - 122,209,106
	Chr7-P1	316F10	chr7: 12,822,528 - 13,028,391
	Chr10-P4	365P10	chr10: 36,903,336 - 37,029,299
	Chr12-P3	90I21	chr12: 43,105,327 - 43,256,924
	Chr14-P4	463A10	chr14: 68,529,221 – 68,704,987
	Chr21-P1	812E1	chr21: 17,645,573 - 17,839,589
Negative Controls	Chr1-Control	379K17	chr1: 219,065,978 - 219,233,134
	Chr3-Control	221E20	chr3: 128,509,052 - 128,695,100
	Chr5-Control	182E4	chr5: 170,105,479 - 170,290,964
	Chr17-Control	304M13	chr17: 15,421,979 - 15,619,583
	Chr19-Control	568L16	chr19: 45,694,980 - 45,871,767
	Chr20-Control	80N12	chr20: 16,775,470 - 16,948,316
