# Supplementary material for: Papillomavirus Genomes Associate with BRD4 to Replicate at Fragile Sites in the Host Genome
Source: PLoS Pathog. 2014 May 15;10(5):e1004117. doi: 10.1371/journal.ppat.1004117 (PMC4022725; doi:10.1371/journal.ppat.1004117)
Supplement: Table S11 — List of siRNAs. (PDF) [file ppat.1004117.s020.pdf]

Supplementary Table 11. SiRNAs used in this study

Target gene (Human)	Company	Cat. No	siRNAs
BRD4	Qiagen	SI03190845	Hs_BRD4_6 FlexiTube siRNA
		SI03238361	Hs_BRD4_7 FlexiTube siRNA
		SI04235812	Hs_BRD4_8 FlexiTube siRNA
		SI04307009	Hs_BRD4_9 FlexiTube siRNA
CREBBP	Qiagen	SI02622648	Hs_CREBBP_2 FlexiTube siRNA
		SI02633085	Hs_CREBBP_6 FlexiTube siRNA
		SI02633092	Hs_CREBBP_7 FlexiTube siRNA
		SI02633099	Hs_CREBBP_8 FlexiTube siRNA
EP300	Qiagen	SI02622592	Hs_EP300_3 FlexiTube siRNA
		SI02626267	Hs_EP300_7 FlexiTube siRNA
		SI03038259	Hs_EP300_9 FlexiTube siRNA
		SI03078761	Hs_EP300_10 FlexiTube siRNA
KAT5	Qiagen	SI00088053	Hs_HTATIP_2 FlexiTube siRNA
		SI00162981	Hs_HTATIP_5 FlexiTube siRNA
		SI02637383	Hs_HTATIP_6 FlexiTube siRNA
		SI02780897	Hs_HTATIP_8 FlexiTube siRNA
	Dharmacon	LU-006301	ON-TARGETplus KAT5 siRNA
Control	Qiagen	1027280	AllStars Negative Control siRNA
	Dharmacon	D-001810-10-05	ON-TARGETplus Non-targeting Pool
